# Reactive oxygen species‐responsive dual‐targeted nanosystem promoted immunogenic cell death against breast cancer

**DOI:** 10.1002/btm2.10379

**Published:** 2022-08-03

**Authors:** Asmita Banstola, Mahesh Pandit, Ramesh Duwa, Jae‐Hoon Chang, Jee‐Heon Jeong, Simmyung Yook

**Affiliations:** ^1^ College of Pharmacy Keimyung University Daegu South Korea; ^2^ Department of Dermatology, Harvard Medical School Wellman Center for Photomedicine, Massachusetts General Hospital Boston Massachusetts USA; ^3^ College of Pharmacy Yeungnam University Gyeongsan Gyeongbuk South Korea; ^4^ Department of Precision Medicine, School of Medicine Sungkyunkwan University Suwon South Korea

**Keywords:** calreticulin, immune cell infiltration, immune cells recruitment, immunogenic cell death, smart nanosystem

## Abstract

The development of an optimal treatment modality to improve the therapeutic outcome of breast cancer patients is still difficult. Poor antigen presentation to T cells is a major challenge in cancer immunotherapy. In this study, a synergistic immunotherapy strategy for breast cancer incorporating immune cell infiltration, immunogenic cell death (ICD), and dendritic cell (DC) maturation through a reactive oxygen species (ROS)‐responsive dual‐targeted smart nanosystem (anti‐PD‐L1‐TKNP) for the simultaneous release of DOX, R848, and MIP‐3α in the tumor microenvironment is reported. Following local injection, anti‐PD‐L1‐DOX‐R848‐MIP‐3α/thioketal nanoparticle (TKNP) converts tumor cells to a vaccine owing to the combinatorial effect of DOX‐induced ICD, R848‐mediated immunostimulatory properties, and MIP‐3α‐induced immune cell recruitment in the tumor microenvironment. Intratumoral injection of anti‐PD‐L1‐DOX‐R848‐MIP‐3α/TKNP caused significant regression of breast cancer. Mechanistic studies reveal that anti‐PD‐L1‐DOX‐R848‐MIP‐3α/TKNP specifically targets tumor tissue, resulting in maximum exposure of calreticulin (CRT) and HMGB1 in tumors, and significantly enhances intratumoral infiltration of CD4^+^ and CD8^+^ T cells in tumors. Therefore, a combined strategy using dual‐targeted ROS‐responsive TKNP highlights the significant application of nanoparticles in modulating the tumor microenvironment and could be a clinical treatment strategy for effective breast cancer management.

## 1 INTRODUCTION

Dendritic cells (DCs), as professional antigen‐presenting cells (APCs), are crucial in cancer immunotherapy by presenting antigens to cytotoxic T cells.^1^ Although DCs are effective APCs, insufficient Toll‐like signaling leads to poor antigen presentation to T cells. Immunoadjuvants such as Toll‐like receptor (TLR) agonists induce Toll‐like signaling and are vital in DC maturation.^2^ Resiquimod (R848), a TLR7/8 agonist which causes DC maturation, has a potent antitumor effect in skin cancer and advanced leukemia patient.^3^

Numerous clinical trials have investigated the immunogenic cell death (ICD) phenomenon exerted by chemotherapeutic agents.^4^ Recent studies have demonstrated that anticancer agents such as oxaliplatin and doxorubicin (DOX) alter the tumor microenvironment and cause ICD of tumor cells.^5^ ICD is characterized by calreticulin (CRT) exposure on the cell surface, high‐mobility group box 1 (HMGB1) release from the cell nucleus, and adenosine triphosphate (ATP) secretion, which provides an “eat me” signal for cytotoxic T‐cell activation.^6^ Despite its significant therapeutic benefit, ICD has failed to produce a robust anticancer effect because of the nonspecificity or low‐dose distribution in the tumor tissue encountered with the free chemotherapeutic drugs.^7^

Despite the large application of immunoadjuvant and chemotherapeutic agents, cancer immunotherapy still faces challenges because of the insufficient infiltration of immune cells in the tumor microenvironment.^8^ Chemokine therapy can address this issue as it enhances immune cell recruitment in the tumor microenvironment.^9^ Chemokines such as macrophage inflammatory protein‐3 alpha (MIP‐3α) bind with the CCR6 receptor expressed on DCs and cause DC recruitment at the inflammation site.^10^

Combination therapy with chemotherapeutic agents, immunoadjuvants, and chemokines has a robust antitumor effect in cancer immunotherapy.^11^ However, combination therapy has a poor pharmacokinetic profile, nonspecificity, and the activation of pro‐inflammatory cascade leading to serious side effects.^12^

**FIGURE 1:**
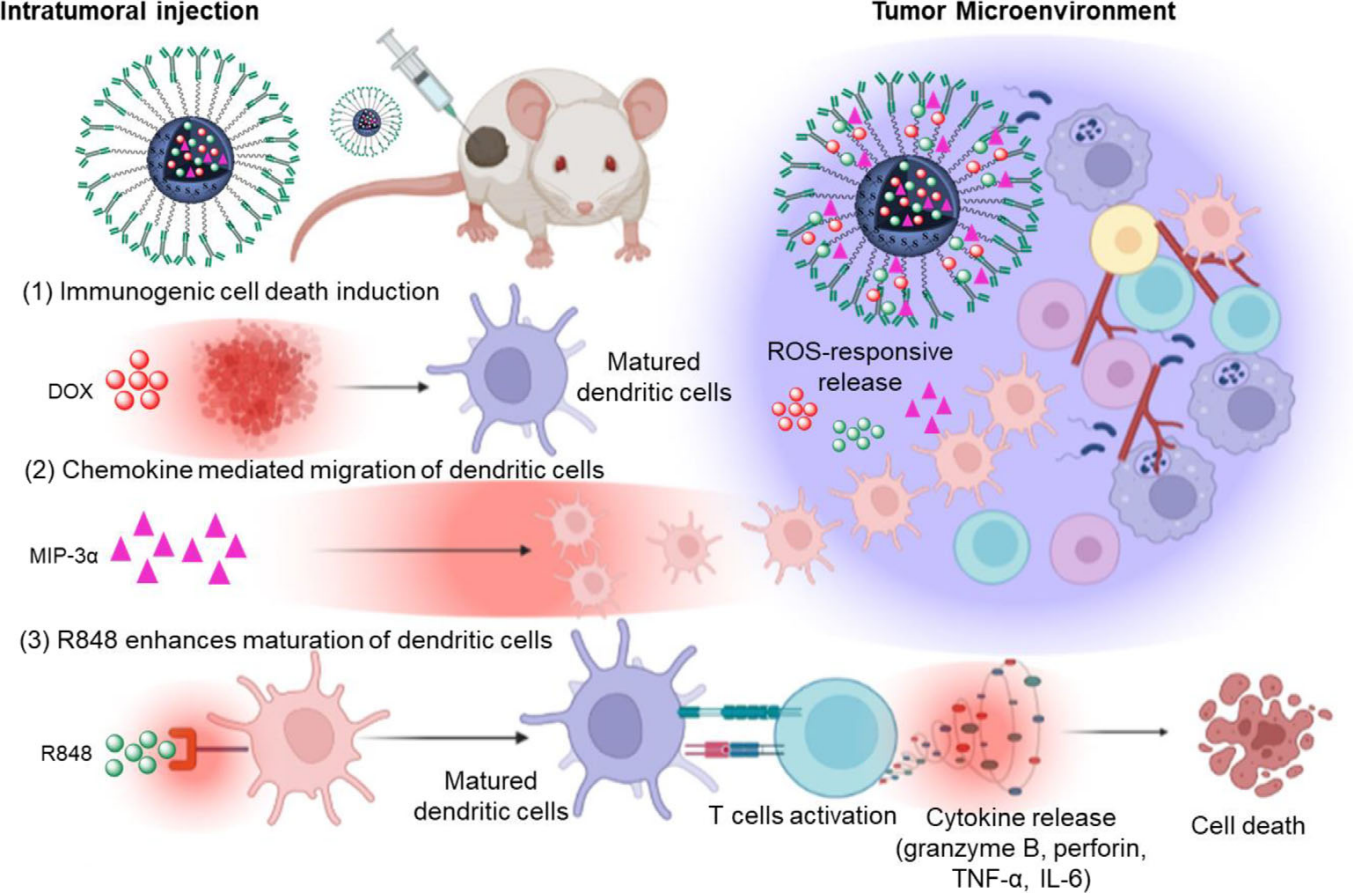
Schematic illustration depicting the anti‐PD‐L1‐DOX‐R848‐MIP‐3α/TKNP‐mediated activation of cellular immunity against breast cancer. A mouse xenograft model was developed to evaluate the therapeutic efficacy of anti‐PD‐L1‐DOX‐R848‐MIP‐3α/TKNP following intratumoral administration. In the tumor microenvironment, degradation of TKNP occurs due to the high ROS level. Release of DOX from TKNP causes ICD, chemokines enhance the migration of dendritic cells, and R848 causes maturation of dendritic cells. Thus, the synergy between the ICD phenomenon and the migration and maturation of dendritic cells results in the activation of T cells. Meanwhile, activated T cells release cytokine causing tumor eradication

To address this issue, tumor microenvironment‐responsive nanoparticles were rationally designed for combination chemoimmunotherapy.^13^, ^14^ Benefiting from the high difference in reactive oxygen species (ROS) level between normal and tumor cells (100‐fold), we constructed ROS‐responsive thioketal nanoparticles (TKNPs) for better tumor specificity.^15^ Various ROS‐responsive moieties such as thioketals, peroxalates, and selenium‐containing linkages are being used for constructing ROS‐responsive nanoparticles.^16^ However, thioketal linkage was employed in our study for constructing ROS‐responsive nanoparticles because of its better stability to enzymatic degradation.^17^ Furthermore, we sought to develop a dual‐targeted nanosystem by conjugating anti‐PD‐L1 antibodies on the ROS‐responsive nanoparticle surface to enhance cellular internalization. Our engineered nanosystem was further co‐encapsulated with DOX, R848, and MIP‐3α to elicit tumor‐specific immune response. Our approach of co‐encapsulation preserves the immunological activities of chemotherapeutic agents, immunoadjuvants, and chemokines, thereby providing maximal therapeutic outcomes.^18^ In the high ROS condition of the tumor microenvironment, TKNPs convert into hydrophilic sulfoxides and sulfones, enabling the release of therapeutic cargos in the tumor microenvironment.^19^ We believe that our optimized nanosystem will prime CD8^+^ T cells and demonstrate a strong antitumor effect against local and metastatic tumors, thereby having a great impact on the development of personalized cancer immunotherapy (Figure 1).

## 2 RESULTS

### 2.1 Synthesis and characterization of ROS‐responsive thioketal polymer and dopamine‐conjugated poly(ethylene‐alt‐maleic acid)

**FIGURE 2:**
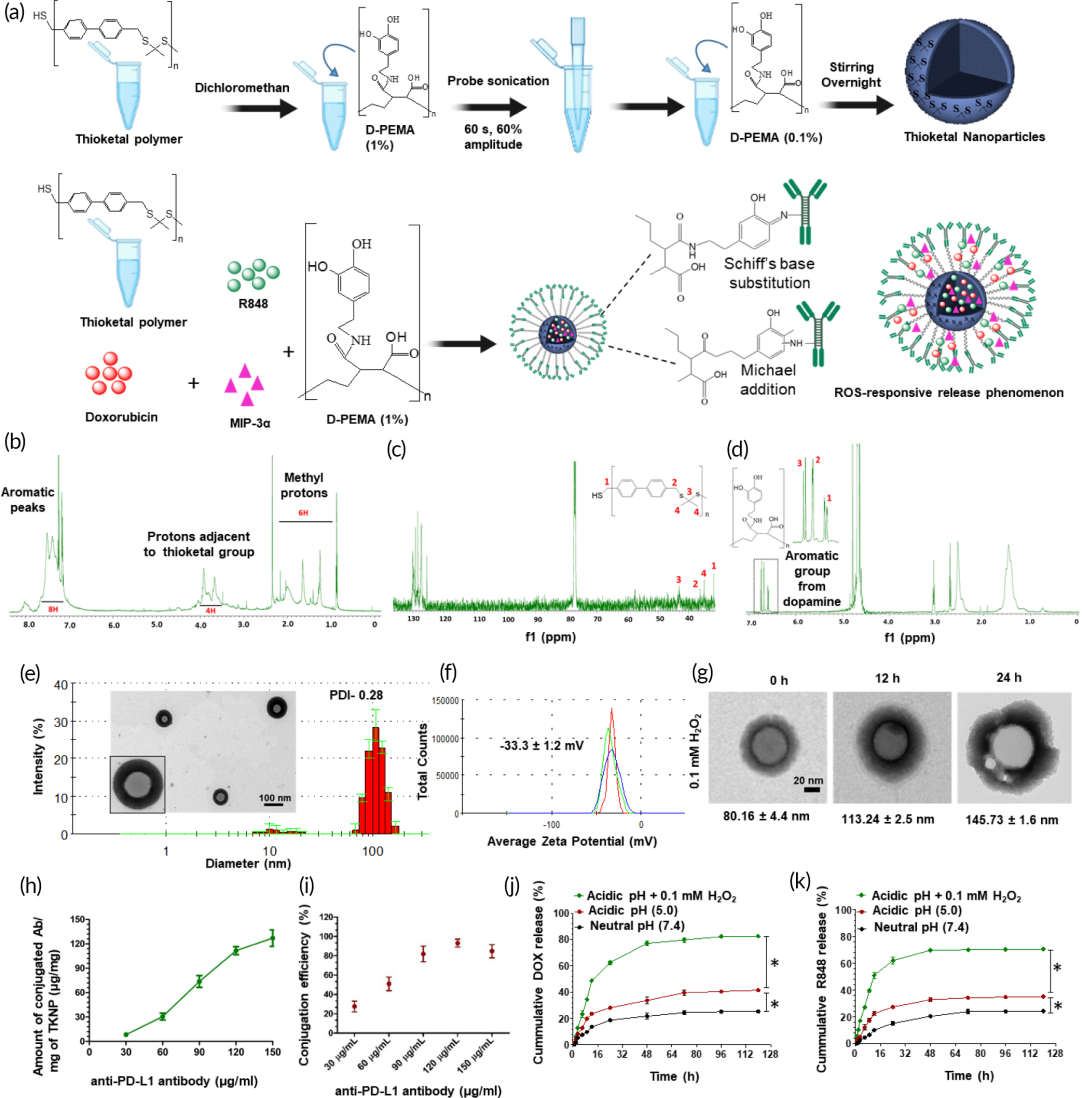
(a) Synthetic scheme for the synthesis of ROS‐responsive immunomodulatory nanosystem. (b) ^1^H NMR and (c) ^13^C NMR of thioketal polymer. (d) ^1^H NMR of D‐PEMA. (e) Hydrodynamic diameter, TEM image, and zeta potential (f) measurement of TKNP. (g) Morphological characterization showing the degradation of TKNP after incubation with 0.1 mM H_2_O_2_. (h) Amount of anti‐PD‐L1 antibody conjugated on TKNP. (i) CE of different amounts of anti‐PD‐L1 antibody conjugated on TKNP. The release profile of DOX (j) and R848 (k) from anti‐PD‐L1‐DOX/R848 at an acidic pH (5.0), acidic pH (5.0) + 0.1 mM H_2_O_2,_ and neutral pH (7.4). Data are expressed as the mean ± SD

Thioketal group was introduced in the stepwise polymerization reaction of 4,4′‐bis(mercaptomethyl)biphenyl and 2,2‐dimethoxypropane (DMP). Figure 2b shows the ^1^H‐nuclear magnetic resonance (NMR; CDCl_3_) of the thioketal polymer suggesting the 6H (methyl group) and 4H group further depicted the proton adjacent to the thioketal group. The proton peak at approximately 7–8 ppm suggested an aromatic ring. Figure 2c shows the ^13^C‐NMR results of thioketal polymer with δ ppm @ 32.01, 35.23, 36.12, and 43.26. ^1^H‐NMR (Figure 2d) further demonstrates the successful construction of dopamine‐conjugated poly(ethylene‐alt‐maleic acid) (DPEMA). The ^1^H‐NMR (D_2_O) of DPEMA showed a peak at δ (ppm) 6.61 (^1^H), 6.69 (^1^H), and 6.76 (^1^H), suggesting the presence of aromatic proton and carbon, confirming the successful synthesis of DPEMA.

### 2.2 Construction and characterization of DOX, MIP‐3α, and R848‐laden TKNP

The DOX, MIP‐3α, and R848‐laden TKNP were prepared using an emulsion–solvent evaporation technique (Figure 2a). Transmission electron microscopy (TEM) demonstrated that the synthesized TKNP has a spherical morphology with a particle size of 80.2 ± 4.4 nm. Dynamic light scattering (DLS; Brookhaven Instruments Corporation, Holtsville, NY) suggested that the TKNP hydrodynamic diameter was 94.5 ± 1.4 nm, while the polydispersity indices (PDI) and average zeta potential were 0.28 and 36.9 ± 1.9 mV, respectively (Figure 2e). The detailed result of optimization of hydrodynamic diameter, loading capacity (LC), and encapsulation efficiency (EE) with different mass ratios for the synthesis of DOX, MIP‐3α, and R848‐loaded TKNP is described in Supporting Information. The optimized thioketal polymer, DOX, R848, and MIP‐3α in the mass ratio of 10:1:0.5:0.04, respectively, was selected which showed a hydrodynamic diameter of 101.0 ± 1.5 nm, PDI of 0.26, and zeta potential of −33.3 ± 1.2 mV as measured using DLS (Figure 2e,f). The LC and EE of DOX were 3.6 ± 0.1% and 35.9 ± 1.2%, respectively, as measured using a fluorescence spectrometer (Figure S1). The LC and EE of R848 were 3.8 ± 0.3% and 76.8 ± 6.9%, respectively, as measured using UV spectroscopy (Figure S1). Quantification of the MIP‐3α amount using a pierce bicinchoninic acid (BCA) protein assay kit showed an LC of 0.4 ± 0.3% and EE of 91.9 ± 9.6% (Figure S1).

### 2.3 ROS‐responsive disassembly of TKNP

TEM demonstrated ROS‐responsive degradation of nanoparticles following exposure to 0.1 mM H_2_O_2_. The particle size was 80.16 ± 4.4 nm at 0 h, 113.24 ± 2.5 nm at 6 h, and 145.73 ± 1.6 nm at 12 h (Figure 2g).

### 2.4 Tailoring of anti‐PD‐L1 antibody on TKNP using DPEMA

DPEMA provides an active site for the adhesion of anti‐PD‐L1 antibody to the TKNP surface via Schiff's base substitution or Michael addition reaction. The particle size of the optimized anti‐PD‐L1 antibody anchored on TKNP was 103.3 ± 3.0 nm, while the PDI was 0.28, and the zeta potential was −10.5 ± 0.3 mV (Figure S3). The amount of anti‐PD‐L1 antibody adhered on the surface of 1 mg TKNP was 111.6 ± 8.5 μg (Figure 2h), and the conjugation efficiency (CE) was 93.0 ± 7.1% (Figure 2i). Stability results also suggested that anti‐PD‐L1‐DOX‐R848‐MIP‐3α/TKNP was highly stable in PBS for 7 days with no change in the particle size and the particle size was similar (104.1 ± 3.4 nm) to PDI‐0.29 (Figure S4). The Supporting Information describes detailed results regarding optimization of the anti‐PD‐L1 antibody amount conjugated on TKNP (Figure S5).

### 2.5 Oxidative stress‐mediated release of DOX and R848

DOX and R848 are released from the TKNP surface owing to the oxidative stress‐mediated degradation of TKNP (Figure 2j,k). Following incubation for 120 h at an acidic pH, the release of DOX and R848 was approximately 41% and 35%, respectively. Incubation at neutral pH for 120 h caused an approximately 25% and 24% release of DOX and R848, respectively. However, following incubation with 0.1 mM H_2_O_2_ for up to 120 h, the release of DOX and R848 was approximately 82% and 70%, respectively. Cumulative release of DOX and R848 in response to oxidative stress was attributed to nanoparticle degradation.

### 2.6 Cellular internalization study of nanoparticles and DOX in 2D and 3D models

**FIGURE 3:**
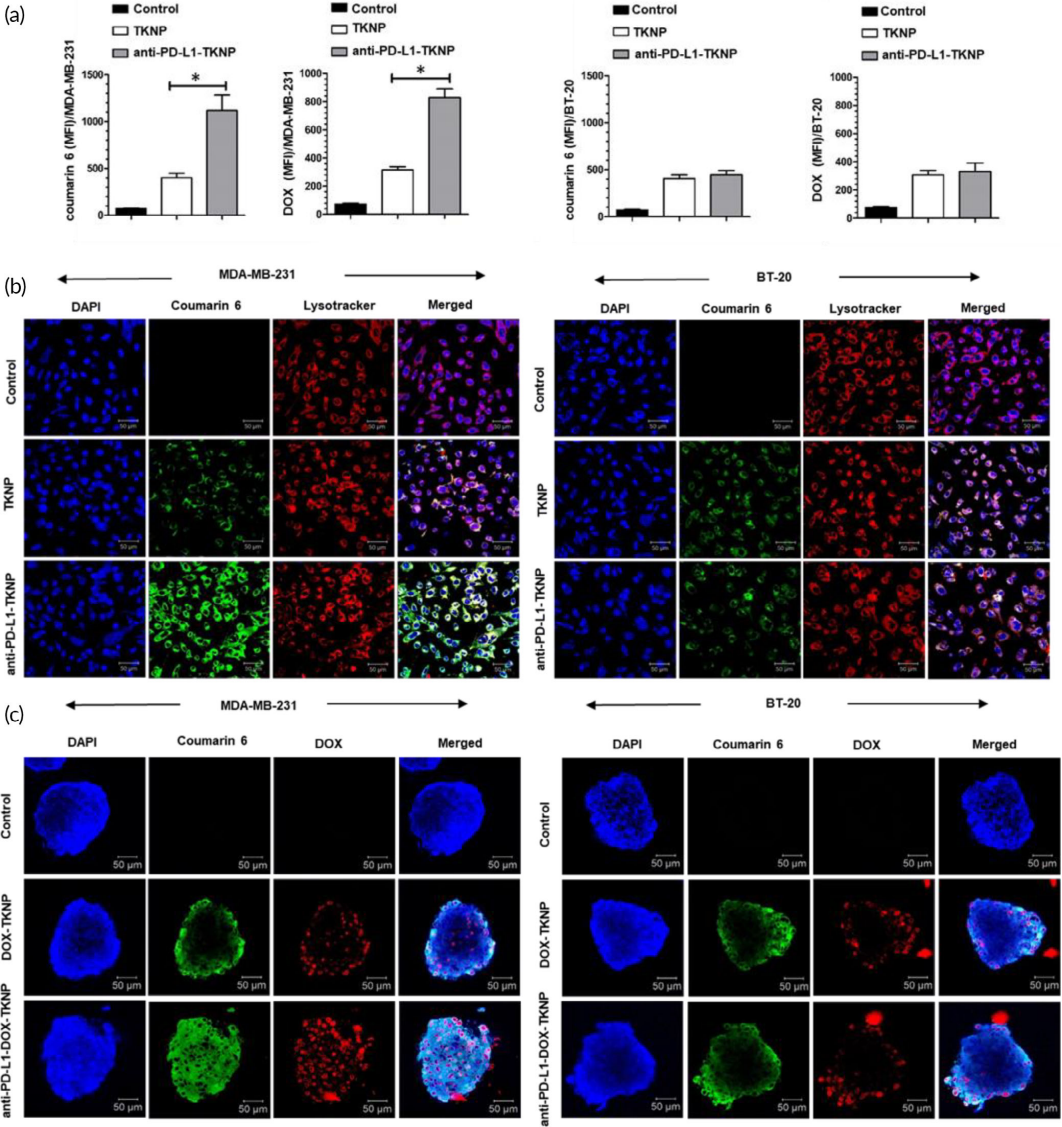
(a) FACS analysis for the intracellular uptake of nanoparticles and DOX following anti‐PD‐L1‐TKNP treatment in MDA‐MB‐231 and BT‐20 cell lines. (b) CLSM images showing cellular internalization of nanoparticles in MDA‐MB‐231 and BT‐20 cell lines after anti‐PD‐L1‐TKNP treatment. (c) CLSM images showing cellular internalization of nanoparticles and DOX in MDA‐MB‐231 and BT‐20 cell lines in the 3D model after anti‐PD‐L1‐DOX‐TKNP treatment

When compared to TKNP alone, anti‐PD‐L1‐TKNP treatment resulted in increased internalization of nanoparticles (2.7‐fold) and DOX (2.6‐fold) in the MDA‐MB‐231 cell line, as determined using fluorescence‐activating cell sorting (FACS) analysis. However, there was no significant difference in the cellular internalization of nanoparticles and DOX in nontargeted and anti‐PD‐L1‐targeted TKNP in the BT‐20 cell line (Figure 3a).

Confocal laser scanning microscopy (CLSM) demonstrated that compared to TKNP alone, anti‐PD‐L1‐TKNP showed increased cellular internalization of TKNP (strong green fluorescence) in the MDA‐MB‐231 cell line. Furthermore, in the BT‐20 cell line, there was no significant difference in cellular internalization between TKNP and anti‐PD‐L1‐TKNP (Figure 3b).

Internalization of nanoparticles and DOX was further evaluated by generating a 3D model of MDA‐MB‐231 and BT‐20 cell lines (Figure 3c). CLSM demonstrated that compared to the control, DOX/TKNP showed significant internalization of DOX (red fluorescence) and nanoparticles (green fluorescence) both in MDA‐MB‐231 and BT‐20 cell lines. However, following anti‐PD‐L1‐DOX/TKNP treatment, MDA‐MB‐231 showed significant internalization of nanoparticles and DOX compared to DOX/TKNP alone. However, there was no significant difference in the cellular internalization of DOX and nanoparticle between targeted and nontargeted nanoparticles in MDA‐MB‐231 and BT‐20 cell lines. Furthermore, the competitive receptor binding assay showed decrease in fluorescence intensity of DOX and coumarin‐6 following 4 h incubation with anti‐PD‐L1 antibody pretreatment + anti‐PD‐L1‐DOX‐TKNP group compared to anti‐PD‐L1‐DOX‐TKNP (Figure S6). Thus, this study also further highlights the PD‐L1 receptor‐mediated cellular internalization of anti‐PD‐L1‐DOX‐TKNP.

### 2.7 ICD phenomenon

**FIGURE 4:**
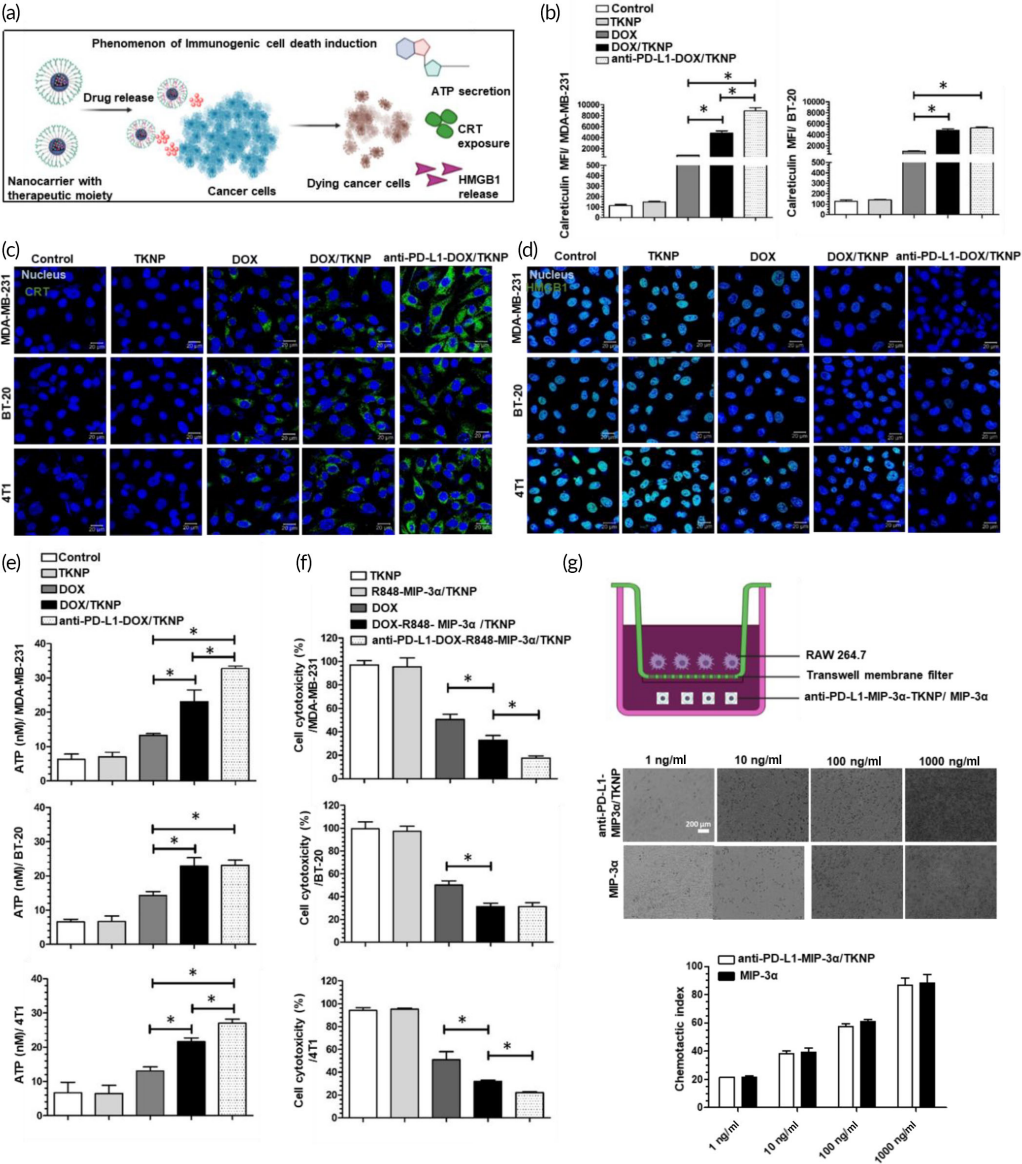
(a) Scheme showing immunogenic cell death phenomenon. (b) Flow cytometry analysis of CRT exposure in MDA‐MB‐231 and BT‐20 cell lines after treatment with TKNP, DOX, DOX/TKNP, and anti‐PD‐L1‐DOX/TKNP. CLSM for the evaluation of CRT exposure on the cell surface (c) and HMGB1 release (d) in MDA‐MB‐231, BT‐20, and 4T1 cell lines following treatment with TKNP, DOX, DOX/TKNP, and anti‐PD‐L1‐DOX/TKNP. (e) Determination of extracellular ATP secretion in MDA‐MB‐231, BT‐20, and 4T1 cell lines following treatment with TKNP, DOX, DOX/TKNP, and anti‐PD‐L1‐DOX/TKNP. (f) In vitro cell cytotoxic effect in MDA‐MB‐231, BT‐20, and 4T1 cell lines after treatment with TKNP, DOX, DOX‐R848‐MIP‐3α/TKNP, and anti‐PD‐L1‐DOX‐R848‐MIP‐3α/TKNP. (g) Chemotaxis of MIP‐3α and anti‐PD‐L1‐MIP‐3α/TKNP after incubation with various concentrations of MIP‐3α using a transwell migration assay

Next, we investigated CRT exposure, HMGB1 release, and extracellular secretion of ATP following treatment with different formulations in MDA‐MB‐231, BT‐20, and 4T1 cell lines (Figure 4a). FACS analysis demonstrated that compared to the control, DOX treatment caused an increment in CRT exposure in both MDA‐MB‐231 (6.9‐fold; *p* < 0.001) and BT‐20 (7.7‐fold; *p* < 0.001) cell lines (Figure 4b). Incubation with DOX/TKNP further increased CRT expression in both MDA‐MB‐231 (6.1‐fold; *p* < 0.001) and BT‐20 (4.9‐fold; *p* < 0.001) cell lines compared to free DOX. CRT expression significantly increased following anti‐PD‐L1‐DOX/TKNP treatment (1.8‐fold; *p* < 0.01) in the MDA‐MB‐231 cell line compared to the DOX‐TKNP‐treated group. However, there was no significant difference in CRT expression in the BT‐20 cell line between nontargeted and targeted groups.

Qualitative analysis of CRT exposure following treatment with different formulations in MDA‐MB‐231, BT‐20, and 4T1 cells was performed using CLSM (Figure 4c). In accordance with the FACS result, CLSM showed increased exposure of CRT (strong green fluorescence surrounding the cell nucleus) in MDA‐MB‐231 and 4T1 cells following anti‐PD‐L1‐DOX/TKNP treatment compared to DOX/TKNP. However, there was no significant difference in CRT exposure between nontargeted and targeted groups in the BT‐20 cell line.

HMGB1 expression was observed in MDA‐MB‐231, BT‐20, and 4T1 cell lines (Figure 4d) following treatment with various formulations. In comparison to the BT‐20 cell line, MDA‐MB‐231 and 4T1 cell lines treated with anti‐PD‐L1‐DOX/TKNP showed the highest release of HMGB1 from the nucleus. Similarly, DOX/TKNP showed enhanced release of HMGB1 from the cell nucleus in MDA‐MB‐231, BT‐20, and 4T1 cell lines compared to free DOX, observed as decreased green fluorescence (HMGB1) in the cell nucleus. However, in the BT‐20 cell line, there was no change in the HMGB1 release between targeted and nontargeted groups. Among all cell lines, maximum CRT exposure was observed in the MDA‐MB‐231 cell line after treatment with anti‐PD‐L1‐DOX/TKNP.

Extracellular secretion of ATP was measured using an ATP assay kit (Figure 4e). Compared to DOX, DOX/TKNP showed significantly increased secretion of ATP in MDA‐MB‐231 (23.1 ± 5.8 nM; *p* < 0.05), BT‐20 (22.8 ± 4.2 nM; *p* < 0.01), and 4T1 (21.6 ± 1.9 nM; *p* < 0.01) cell lines. ATP levels significantly increased following anti‐PD‐L1‐DOX/TKNP treatment in MDA‐MB‐231 (32.7 ± 1.2 nM; *p* < 0.05) and 4T1 (27.1 ± 2.0 nM; *p* < 0.05) cell lines compared to DOX/TKNP treatment. However, there was no difference in extracellular ATP secretion level in the BT‐20 cell line between DOX/TKNP‐treated (22.8 ± 4.2 nM) and anti‐PD‐L1‐DOX/TKNP‐treated (23.2 ± 2.5 nM) groups.

### 2.8 Cell cytotoxicity assay

Next, we assessed the cell cytotoxic effect after treatment with different formulations in MDA‐MB‐231, BT‐20, and 4T1 cell lines (Figure 4f). No cytotoxic effect was observed following TKNP treatment in all cell lines suggesting nontoxicity of the carrier. Similarly, R848‐MIP‐3α/TKNP treatment did not cause a cytotoxic effect in MDA‐MB‐231 (95.5 ± 13.0%), BT‐20 (97.3 ± 7.8%), and 4T1 (95.2 ± 1.6%) cell lines indicating a nonimmunogenic effect in the in vitro system. However, DOX‐R848‐MIP‐3α/TKNP treatment caused a significant cytotoxic effect in MDA‐MB‐231 (32.9 ± 6.6%; *p* < 0.05), BT‐20 (31.3 ± 5.2%; *p* < 0.05), and 4T1 (31.9 ± 2.0%; *p* < 0.05) cell lines compared to free DOX. Additionally, anti‐PD‐L1‐DOX‐R848‐MIP‐3α/TKNP treatment caused a significant cytotoxic effect in MDA‐MB‐231 (17.5 ± 3.4%; *p* < 0.05) and 4T1 (22.1 ± 1.4%; *p* < 0.01) cell lines compared to DOX‐R848‐MIP‐3α/TKNP treatment. However, there was no significant difference in cytotoxic effect in the BT‐20 cell line between targeted and nontargeted groups.

### 2.9 Chemotaxis assay

We further evaluated the chemotactic activity of MIP‐3α after co‐encapsulation inside anti‐PD‐L1‐TKNP (Figure 4g). A chemotactic effect was observed by incubating anti‐PD‐L1‐MIP‐3α/TKNP in the lower chamber of the transwell plate. Anti‐PD‐L1‐MIP‐3α/TKNP was able to attract macrophages compared to free MIP‐3α in a concentration‐dependent manner. The maximum chemotactic activity was observed at 1000 ng/ml anti‐PD‐L1‐MIP‐3α/TKNP, similar to that of free MIP‐3α, suggesting that MIP‐3α remained active after co‐encapsulation in anti‐PD‐L1‐TKNP.

### 2.10 Tumor retention assay

**FIGURE 5:**
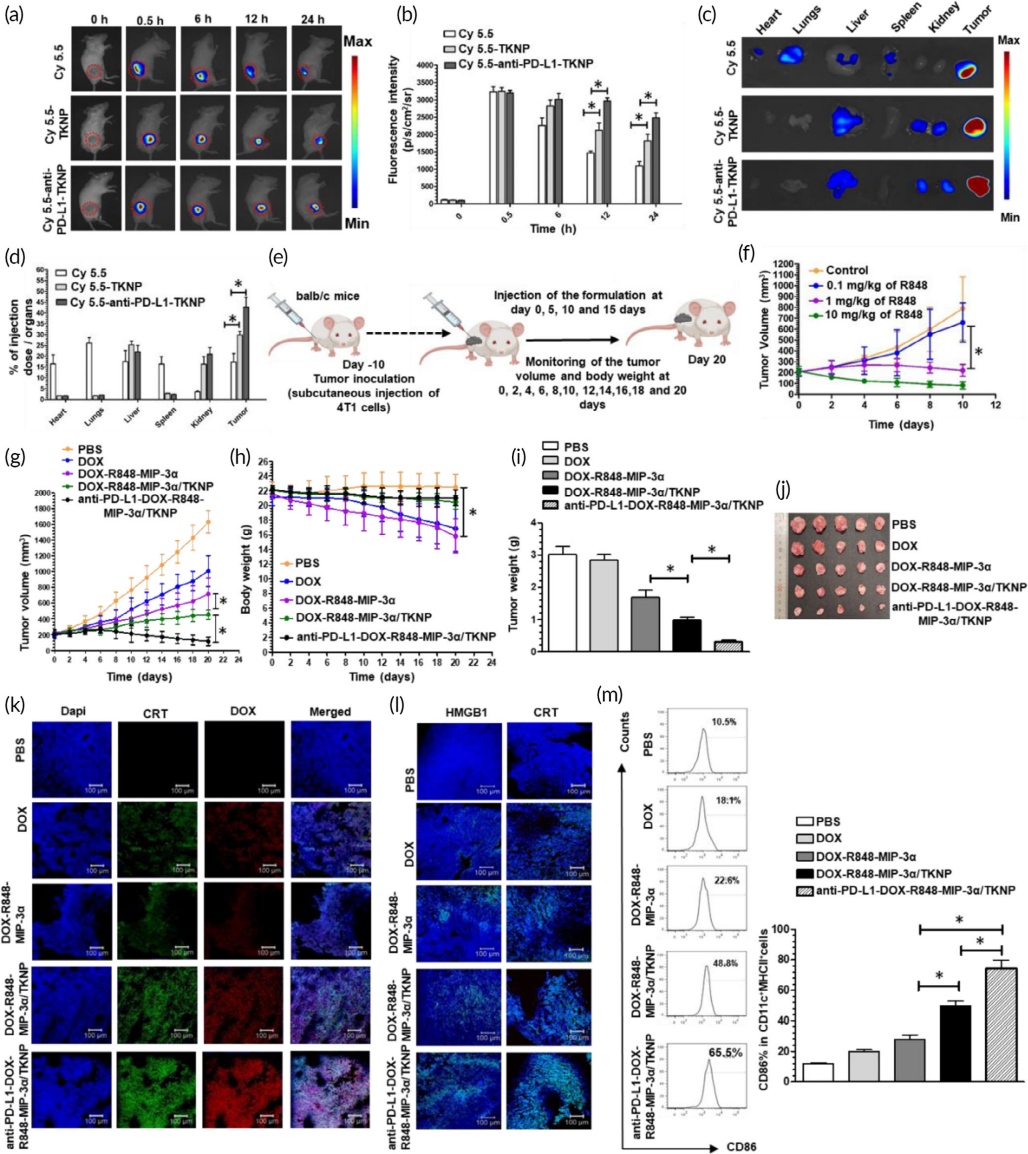
In vivo biodistribution study of Cy5.5 loaded TKNP and anti‐PD‐L1‐TKNP in 4T1 tumor xenograft‐bearing balb/c mice following intratumoral injection and (a) time‐dependent imaging up to 24 h and their quantitative fluorescence intensity analysis (b). (c) Ex vivo imaging and quantitative analysis (d) of their fluorescence intensity distributed in vital organs after 24 h. (e) In vivo antitumor study design. (f) Preliminary antitumor study for dose optimization of R848. Tumor volume (g) and body weight (h) of 4T1 tumor‐bearing mice after the administration of PBS, DOX, DOX‐R848‐MIP‐3α, DOX‐R848‐MIP‐3α/TKNP, and anti‐PD‐L1‐DOX‐R848‐MIP‐3α/TKNP as indicated (*n* = 6). Tumor weight (i) and tumor image (j) of the mice after the sacrifice on day 20. (k) Immunofluorescence staining of tumor sections for the determination of CRT exposure and internalization of DOX after the sacrifice of mice on day 15. (l) Immunofluorescence staining of tumor sections for the determination of CRT exposure and HMGB1 release after the sacrifice of mice on day 20 following treatment with PBS, DOX, DOX‐R848‐MIP‐3α, DOX‐R848‐MIP‐3α/TKNP, and anti‐PD‐L1‐DOX‐R848‐MIP‐3α/TKNP as indicated (*n* = 6). (m) FACS analysis for the evaluation of DC maturation in lymph nodes. Percentages of mature DCs (CD86% in CD11c^+^ MHCII^+^ cells)

To evaluate tumor retention and targeting ability of TKNP and anti‐PD‐L1‐TKNP, In Vivo Imaging System (IVIS) was used, and tumor retention of the nanoparticles was evaluated at 0, 0.5, 6, 12, and 24 h. Nanoparticles were labeled with an equivalent concentration of Cy5.5. The tumor retention assay demonstrated that anti‐PD‐L1‐TKNP displayed longer retention in the tumor zone up to 12 h (1.4‐fold; *p* < 0.05) and 24 h (1.3‐fold; *p* < 0.05) compared to the TKNP alone (Figure 5a,b). Maximum retention of targeted nanoparticles in the tumor zone compared to nanocarrier alone is attributed to the PD‐L1 receptor‐mediated endocytosis compared to the enhanced permeability and retention effect alone.

Subsequently, we sacrificed the mice at 24 h to perform ex vivo imaging of the vital organs. Superior retention in the tumor zone was obtained following anti‐PD‐L1‐TKNP treatment (43.7 ± 7.8%) compared to TKNP (29.8 ± 2.8%) alone (Figure 5c,d). There was no accumulation in vital organs such as the heart and lungs, suggesting nontoxicity of the nanoparticles.

### 2.11 Antitumor study

Encouraged by the superior tumor retention and ICD phenomenon, we subsequently evaluated the antitumor efficacy of anti‐PD‐L1‐DOX‐R848‐MIP‐3α/TKNP following intratumoral injection for 20 days (Figure 5e). Our preliminary dose optimization antitumor study demonstrated that 1 mg/kg equivalent weight of R848 showed a significant antitumor effect with a reduced tumor volume (220.7 ± 55.1 mm^3^; *p* < 0.05) compared to the PBS‐treated group (790.6 ± 292.2 mm^3^; Figure 5f). Additionally, we observed a significant increment in the serum IL‐6 level (140.4 ± 34.1 pg/ml; *p* < 0.01) following treatment with 1 mg/kg equivalent weight of R848 compared to free PBS (Figure S10). Thus, we selected 1 mg/kg equivalent weight of R848 encapsulated in anti‐PD‐L1‐DOX‐R848‐MIP‐3α/TKNP for further experiments. On day 20, DOX alone caused a reduction in tumor volume (1008.7 ± 195 mm^3^, 1.6‐fold; *p* < 0.001) compared to the control (1632.1 ± 141 mm^3^) (Figure 5g). Furthermore, DOX‐MIP‐3α‐R848 cocktail (716.9 ± 205.8 mm^3^, 1.4‐fold; *p* < 0.05) caused a significant reduction in tumor volume compared to free DOX (1008.7 ± 195 mm^3^). Additionally, encapsulation of DOX‐R848‐MIP‐3α in TKNP (455.4 ± 57 mm^3^, 1.6‐fold; *p* < 0.05) further caused a significant reduction in tumor volume compared to the DOX‐R848 MIP‐3α cocktail (716.9 ± 205 mm^3^). Tumor volume significantly reduced following anti‐PD‐L1‐DOX‐R848‐MIP‐3α/TKNP treatment (115.7 ± 54 mm^3^), which was 1.6‐fold (*p* < 0.01) and 3.9‐fold (*p* < 0.001) higher compared to the DOX‐R848‐MIP‐3α and DOX‐R848‐MIP‐3α/TKNP cocktail, respectively. Body weight was also measured to study the toxicity profile of the different formulations (Figure 5h). There was a significant reduction in body weight in the free DOX and DOX‐R848 MIP‐3α‐treated groups compared to the control. However, body weight was similar to the control in the DOX‐R848‐MIP‐3α/TKNP and anti‐PD‐L1‐DOX‐R848‐MIP‐3α/TKNP‐treated groups. Furthermore, the tumor weight was significantly reduced (Figure 5i) in mice treated with anti‐PD‐L1‐DOX‐R848‐MIP‐3α/TKNP which was further confirmed using the morphological tumor images (Figure 5j).

### 2.12 Investigation of ICD phenomenon, DOX retention, and DC maturation study

After confirming the better anti‐cancer effect of anti‐PD‐L1‐DOX‐R848‐MIP‐3α/TKNP in the in vitro condition, we investigated the ICD phenomenon in dying tumor cells. Generally, in ICD, dead cancer cells are eventually converted into a vaccine for activating cytotoxic T cells against tumor cells.^4^ Thus, we investigated the ICD phenomenon after sacrificing mice on day 15. Tumors were excised from the mice on day 15, and the tumor retention of DOX and DOX‐induced ICD phenomenon was examined by immunofluorescence staining of the tumor section. To determine the ICD on day 15, we measured CRT expression in the tumor tissue (Figure 5k). Compared to other groups, anti‐PD‐L1‐DOX‐R848‐MIP‐3α/TKNP increases CRT exposure (green fluorescence) and provides maximum retention of DOX (red fluorescence) in the tumor tissue. Thus, maximum retention of DOX in the tumor tissue offered by anti‐PD‐L1‐DOX‐R848‐MIP‐3α/TKNP resulted in robust ICD induction efficacy.

Furthermore, we also measured CRT and HMGB1 expression in the tumor section of the mice sacrificed on day 20 to further confirm the ICD (Figure 5l). Anti‐PD‐L1‐DOX‐R848‐MIP‐3α/TKNP caused maximum exposure of CRT and HMGB1 release in the tumor zone which is further confirmed by the strong green fluorescence.

Furthermore, ICD provides an “eat me signal” which eventually leads to DC maturation, thereby causing antigen processing and presentation to T cells.^20^ DCs has a pivotal role in the activation of cellular immunity. However, cellular immunity is impaired by the poor antigen presentation by DCs.^21^ DCs follows TLR signaling pathway and assist in the antigen presentation to T cells.^22^ Immunoadjuvants such as R848 play a significant role in DC maturation. Thus, we further examined the expression of surface molecules such as CD86^+^ on CD11c^+^MHCII^+^ DCs derived from lymph nodes to assess DC maturation using a flow cytometer (Figure 5m). In the DOX‐R848‐MIP‐3α/TKNP‐treated mice, the CD86^+^ ratio on CD11c^+^MHCII^+^ DCs was significantly increased to 49.7 ± 5.5%, which was 1.8‐fold (*p* < 0.01) higher than of DOX‐R848‐MIP‐3α (27.6 ± 5.3%) cocktail. Additionally, maximum increment in the CD86^+^ ratio (74.3 ± 9.6%) on CD11c^+^MHCII^+^ DCs was noticed in anti‐PD‐L1‐DOX‐R848‐MIP‐3α/TKNP‐treated mice, which was 2.7‐fold (*p* < 0.001) and 1.5‐fold (*p* < 0.01) higher than that of DOX‐R848‐MIP‐3α and DOX‐R848‐MIP‐3α/TKNP, respectively. Increased DC maturation is accredited to the tumor microenvironment‐specific release of R848 offered by our nanosystem.

### 2.13 Infiltration of CD4 ^+^ and CD8 ^+^ T cells in the tumor microenvironment

**FIGURE 6:**
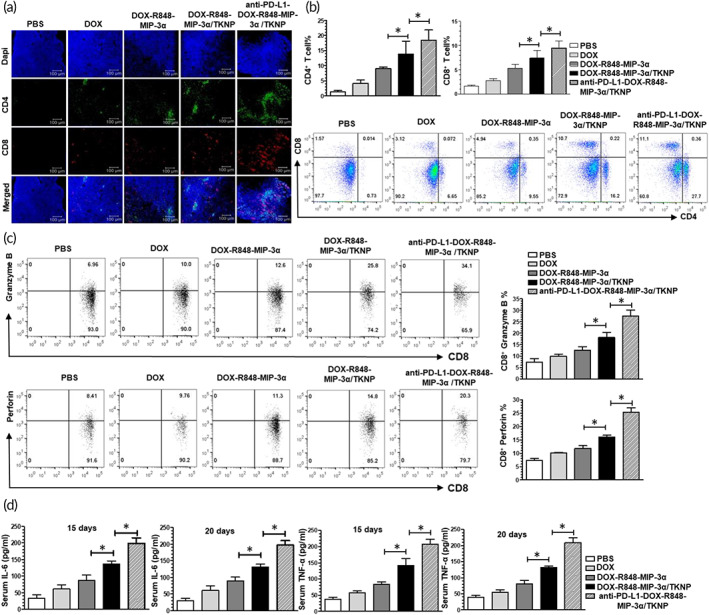
Determination of intratumoral infiltration of immune cells after the sacrifice of mice on day 20. (a) Immunofluorescence staining of tumor sections for the elucidation of CD4^+^ and CD8^+^ T cell expression in the tumor tissue. (b) FACS analysis demonstrating intratumoral infiltration of CD4^+^ and CD8^+^ T cells in 4T1 tumor‐bearing balb/c mice treated with PBS, DOX, DOX‐R848‐MIP‐3α, DOX‐R848‐MIP‐3α/TKNP, and anti‐PD‐L1‐DOX‐R848‐MIP‐3α/TKNP. (c) Flow cytometric examination of intratumoral infiltration of granzyme B^+^CD8^+^ T lymphocytes and perforin^+^CD8^+^ T lymphocytes in 4T1 tumor‐bearing balb/c mice following treatment with PBS, DOX, DOX‐R848‐MIP‐3α, DOX‐R848‐MIP‐3α/TKNP, and anti‐PD‐L1‐DOX‐R848‐MIP‐3α/TKNP. Measurement of serum cytokine and tumor infiltrated cytokine level after 20 days. (d) ELISA analysis for the determination of serum cytokines, IL‐6 and TNF‐α, at days 15 and 20 in mice treated with PBS, DOX, DOX‐R848‐MIP‐3α, DOX‐R848‐MIP‐3α/TKNP, and anti‐PD‐L1‐DOX‐R848‐MIP‐3α/TKNP as indicated (*n* = 6)

It is well reported that increased ICD phenomenon and DC maturation lead to increased intratumoral infiltration of CD4^+^ and CD8^+^ T cells.^23^ Thus, we evaluated CD4^+^ and CD8^+^ T cells expression in the tumor tissue following immunofluorescence staining. CLSM was used to determine the intratumoral infiltration of CD4^+^ and CD8^+^ T cells (Figure 6a). Maximum expression of CD4^+^ and CD8^+^ (green and red fluorescence, respectively) in the tumor section of anti‐PD‐L1‐DOX‐R848‐MIP‐3α/TKNP‐treated mice compared to that in other groups suggested maximum intratumoral infiltration of immune cell.

Similarly, we also evaluated the quantitative expression of intratumoral infiltration of CD4^+^ and CD8^+^ T cells after treatment with different formulations using flow cytometry (Figure 6b). The gating strategy is further provided in Supporting Information (Figure S11). The anti‐PD‐L1‐DOX‐R848‐MIP‐3α/TKNP‐treated group resulted in 9.5 ± 3.2% CD8^+^ and 18.4 ± 7.6% CD4^+^ T lymphocyte infiltration ratio which was 1.2‐fold and 1.3‐fold higher, respectively, than that of the DOX‐R848‐MIP‐3α/TKNP‐treated group. Additionally, the DOX‐R848‐MIP‐3α/TKNP‐treated group showed 7.4 ± 3.4% CD8^+^ T and 13.8 ± 9.7% CD4^+^ T‐cell infiltration ratios which were 1.4‐fold and 1.5‐fold higher, respectively, than that of the DOX‐R848‐MIP‐3α‐treated group. Furthermore, the DOX‐treated group also increased the CD8^+^ (2.8 ± 0.8%) and CD4^+^ (4.1 ± 2.5%) T‐lymphocyte infiltration ratio which was 1.7‐fold and 3.1‐fold higher, respectively, than that of the PBS‐treated group.

### 2.14 Measurement of cytokine level

Activated CD8^+^ T cells secrete cytokines such as granzyme B and perforin which possess a potent antitumor effect. Thus, we measured granzyme B and perforin levels in the tumor tissue using flow cytometry (Figure 6c). DOX‐R848‐MIP‐3α/TKNP caused an increment in granzyme B (18.0 ± 5.1%) and perforin level (16.1 ± 1.4%) which were 1.4‐fold and 1.3‐fold higher, respectively, than those caused by the DOX‐R848‐MIP‐3α cocktail. Furthermore, the granzyme B (27.4 ± 5.9%) and perforin (25.3 ± 3.9%) percentages remarkably increased following anti‐PD‐L1‐DOX‐R848‐MIP‐3α/TKNP treatment which was 1.5‐fold and 1.5‐fold higher, respectively, than that of DOX‐R848‐MIP‐3α/TKNP‐treated groups.

Secreted cytokines play a crucial role in boosting cellular immunity. Therefore, we measured cytokine levels such as IL‐6 and TNF‐α in mice sera at days 15 and 20 using Elisa assay (Figure 6d). Compared to the PBS group, increased serum IL‐6 and TNF‐α levels were observed on days 15 (60.9 ± 22.3 pg/ml and 56.7 ± 12.6 pg/ml) and 20 (60.7 ± 23.3 pg/ml and 54.8 ± 13.8 pg/ml), respectively, in the DOX‐treated group. Compared to the DOX‐R848‐MIP‐3α cocktail, DOX‐R848‐MIP‐3α/TKNP increased the IL‐6 and TNF‐α levels at days 15 (135.7 ± 16.9 pg/ml; 1.6‐fold and 142.5 ± 34.7 pg/ml; 1.7‐fold) and 20 (131.2 ± 14.3 pg/ml; 1.5‐fold and 131.8 ± 8.4 pg/ml; 1.6‐fold), respectively. The incorporation of immunoadjuvant, chemokines, and cytotoxic agents in nanoparticles provides a better anticancer effect, causing an increase in cytokine levels. A more significant increment in the serum IL‐6 and TNF‐α levels were observed in anti‐PD‐L1‐DOX‐R848‐MIP‐3α/TKNP at days 15 (198.7 ± 28.1 pg/ml and 207.8 ± 25.9 pg/ml) and 20 (196.8 ± 22.9 pg/ml and 208.9 ± 25.9 pg/ml), respectively, which is accredited to the PD‐L1‐mediated tumor specificity and ROS‐responsive release of therapeutic cargos.

## 3 DISCUSSION

Clinical data on breast cancer have reflected durable therapeutic response following activation of patient's immune system.^24^ FDA approved atezolizumab for PD‐L1‐positive advanced breast cancer.^25^ However, the proportion of breast cancer patients responding to phase Ib clinical trial of atezolizumab was only 12%.^26^ Major challenges in the immunotherapeutic approach are restricted to an inappropriate dose of cytotoxic drugs in the tumor microenvironment, poor antigen presentation, and poor infiltration of cytotoxic T lymphocytes in the tumor microenvironment.^27^, ^28^ To combat the tumor heterogeneity with activated immunity, and strengthen the efficacy, complex formulations with several agents target different limitations of the therapeutic strategies is necessary.^29^ As a solution to the inappropriate dose of cytotoxic drugs, we used PD‐L1‐targeted and ROS‐responsive nanoparticles enabling the administration of a concentrated dose of chemotherapeutic agents in the tumor site. The poor antigenic presentation was overcome by the nano‐enabled release of R848 in the tumor microenvironment. To address poor infiltration of cytotoxic T lymphocytes, we also introduced chemokine therapy by encapsulating MIP‐3α in our nanoparticles which accelerates immune cell migration in the tumor microenvironment. We have used MDA‐MB‐231^30^, ^31^ and 4T1^31^ cell line for our study as they have been reported for high PD‐L1 expression. However, BT‐20 cell which expresses low PD‐L1^32^ expressions than MDA‐MB‐231 cell line was used for the evaluation of the targeting effect of anti‐PD‐L1‐DOX‐R848‐MIP‐3α/TKNP. Furthermore, we have also mentioned the rationale behind the usage of these cell lines in the manuscript as well.

Despite immunological silence of DOX, several clinical and preclinical evidence has suggested the emergence of DOX‐induced ICD phenomenon.^33^ In a clinical setting, low dose of DOX has been reported to induce ICD. However, off‐target effect and poor selectivity limit the clinical application of DOX‐induced ICD.^6^ In this study, we demonstrate the use of ROS‐responsive and PD‐L1‐targeted nano‐enabled chemotherapy for robust ICD induction in a xenograft 4T1 breast cancer model, an outcome that was not well exerted by free DOX.

Despite immune cell activation via ICD induction, cancer cells still evade their destruction by suppressing immune cells.^34^ To address this issue, we focused on the maturation of antigen‐presenting cells such as DCs via pharmacological activation of TLR using TLR agonist (R848), which further instructs cytotoxic T cells to eradicate tumor cells. R848, which shares a similar structure with the FDA‐approved TLR7/8 agonist imiquimod, is reported to be 100 times more potent.^2^ Systemic administration of R848 exerts antitumor immune responses but a high dose is required for optimal therapeutic effect.^35^ Local injection of R848 combined with chemotherapeutic agents has shown complete tumor regression with long‐term therapeutic effects. Yin et al.^36^ reported that a 3 mg/kg dose of R848 was administered intraperitoneally nine times in 4T1 tumor‐bearing mice model; however, complete tumor eradication was not observed. The tumor volume observed after 24 days was approximately 400 mm^3^. Significant therapeutic effect of R848 is observed only when R848 is administered using a smart drug delivery platform. Our finding suggested that a dual‐targeted nanosystem combined with R848, MIP‐3α, and DOX containing an equivalent dose of R848 (1 mg/kg) injected four times in 4T1 tumor‐bearing mice suppress tumor volume below 100 mm^3^. These results further supported that the nanosystem, following intratumoral administration, improved R848 bioavailability in the tumor microenvironment.

Clinical application of DC‐based vaccine is limited despite activation of tumor‐specific T cells.^37^ Numerous clinical trials have demonstrated the success of DC‐based vaccines in phase I and phase II clinical trial; however, in phase III clinical trial, poor efficacy was observed.^38^ One of the major reasons underlying the poor success rate is poor migration of DCs in the lymph node.^39^ Here, we introduced chemokine therapy to solve the issue regarding poor infiltration of immune cells.^40^ The chemotaxis assay performed in our study demonstrated that MIP‐3α incorporated in our nanosystem caused migration of RAW264.7 macrophages, which demonstrated that poor infiltration of immune cells or antigen‐presenting cells could be solved by introducing chemokines. We also observed the maximum percentage of infiltrated cytokines in the anti‐PD‐L1‐DOX‐R848‐MIP‐3α/TKNP‐treated tumor tissue. Thus, we believed that the nanosystem constructed by incorporating MIP‐3α caused the recruitment of immature DCs in the tumor microenvironment, further increasing cellular immunity against breast cancer.^41^

Local injection provides better T cell priming in the tumor microenvironment compared to systemic injection.^42^ In cancer immunotherapy, local injections utilize tumor cells as its own vaccine and help to exert a systemic immune response against tumor cells.^43^ Additionally, local injection of immune‐stimulating moieties enhances tumor antigen recognition and also ensures access to the cytotoxic T cells infiltrated in the tumor tissue.^44^ In a study by Song et al.,^45^ after the systemic injection of R848‐loaded bismuth selenide nanocage following NIR irradiation to 4T1 tumor‐bearing mice, the amount of CD8^+^ T cells infiltrated in the tumor tissue was below 5%. Thus, in our study, we aimed to increase the percentage of CD8^+^ T cells in the tumor tissue by locally injecting anti‐PD‐L1‐DOX‐R848‐MIP‐3α/TKNP in 4T1 tumor‐bearing mice. We demonstrated maximum priming of T cells in the tumor tissue with CD8^+^ T cells (9.5 ± 3.2%) and CD4^+^ T cells (18.4 ± 7.6%) suggesting local injection of R848 embedded in a smart nanosystem increases cytotoxic activities of T cells.

Due to maximal tumor deposition, significant active and passive targeting, and ROS‐responsive release properties, TKNP combined with immunotherapeutic moiety caused maximal tumor eradication with low side effects. The anti‐PD‐L1‐DOX‐R848‐MIP‐3α/TKNP has significant potential to enhance the prognosis of patients with refractory tumor in a clinical setting and has a wide range of clinical applications. Additionally, targeted delivery of immunoadjuvants to endogenous immune cells and priming of immune cells in the tumor microenvironment highlighted the potential of this strategy for additional investigation in overcoming immunological tolerance. This study also further highlighted the scope of immune checkpoint blockade therapy which could be due to the dissociation of an anti‐PD‐L1 antibody from our nanocarrier. In addition, the therapeutic payload encapsulated in our nanosystem can function for synergistic effect as high infiltration of DCs in the tumor microenvironment due to MIP‐3α can boost the therapeutic effect of R848 on triggering DC maturation. Furthermore, we can also broaden the scope of this study by studying the interaction of these active drugs with the several cell type present in the tumor microenvironment.

## 4 MATERIALS AND METHODS

### 4.1 Synthesis and characterization of ROS‐responsive thioketal polymer

Thioketal polymer was constructed using an acetal exchange reaction following stepwise polymerization. Briefly, 4,4′‐bis(mercaptomethyl)biphenyl (16 mg/mL; Sigma‐Aldrich Corp., St. Louis, MO) dissolved in toluene (Sigma‐Aldrich Corp.) was further mixed with 2,2‐DMP (250 μl; Sigma‐Aldrich Corp.). Next, the mixture was stirred at 75°C and *p*‐toluenesulfonic acid (2 mg; Sigma‐Aldrich Corp.) dissolved in ethyl acetate (250 μl) to initiate the reaction. After 1 h of reaction, a mixture of DMP (500 μl) in toluene (10 ml) was kept at an interval of every 30 min for 10 h for polymerization. Precipitation with cold hexane (Sigma‐Aldrich Corp.) was performed to obtain thioketal polymer. Characterization of thioketal polymer was performed using NMR (JEOL NMR, ECZ 500 R; JEOL Ltd., Tokyo, Japan).

### 4.2 Construction and characterization of DPEMA

PEMA was obtained by the hydrolysis of PEMAnh (poly[ethylene‐alt‐maleic anhydride]; Sigma‐Aldrich Corp.). The insoluble PEMAnh was converted to soluble PEMA by mixing PEMAnh (17 mg/ml) in deionized water, and the solution was heated at 60°C for 6 h. Lyophilization was performed to obtain PEMA powder. A nucleophilic addition reaction for 18 h was performed to obtain DPEMA following the reaction between PEMA and dopamine. The organic solvent was removed via dialysis and obtained DPEMA was analyzed using NMR.

Construction and characterization of DOX, MIP‐3α, and R848‐laden TKNP. DOX/R848/MIP‐3α‐co‐loaded TKNP were prepared using the emulsion solvent evaporation technique.^46^ Briefly, thioketal polymer (1 mg) was dissolved in dichloromethane (5 ml) containing DOX (Zhejing Hisum Co, Zhejiang, China), R848 (MedChemExpress, MCE, NJ), and MIP‐3α (Biolegend, San Diego, CA) in the mass ratio of 10:1:0.5:0.04, respectively. The Supporting Information further describes the optimization of nanoparticles prepared with a different mass ratio of thioketal polymer, DOX, R848, and MIP‐3α. DPEMA (1% [w/v], 5 ml) was added. Next, the solution was sonicated (Sonics and Materials, Inc., Newtown, CT) for 60 s at 60% amplitude using a probe sonicator. Furthermore, DPEMA (0.1% [w/v], 5 ml) was added following sonication, and stirring was continued overnight. Repeated purification was carried out the following centrifugation at 15,000 × *g* for 10 min to obtain DOX‐R848‐MIP‐3α‐co‐loaded TKNP.

Characterization of DOX/R848/MIP‐3α‐co‐loaded TKNP was done by DLS to measure hydrodynamic diameters, zeta potentials, and PDI. Similarly, TEM was used to trace the morphology and particle size. The DOX amount was assessed based on the calibration curve obtained from a fluorescence spectrometer with emission at 580 nm and excitation at 480 nm. Similarly, R848 concentration was measured based on the calibration curve obtained from UV spectroscopy by measuring the absorbance at 254 nm. Furthermore, the MIP‐3α content loaded in TKNP was evaluated using Pierce BCA protein assay kit (Thermo Fisher Scientific, Waltham, MA).

Similarly, LC and EE were calculated as follows:

(1) $$\mathrm{LC}\;\left(\%\right)=\left(\frac{\text{Amount of}\frac{\frac{\mathrm{DOX}}{R848}}{\mathrm{MIP}-3\alpha }\;\text{loaded in TKNP}}{\text{The actual amount of TKNP}}\right)\times 100$$

(2) $$\mathrm{EE}\;\left(\%\right)=\left(\;\frac{\text{Actual loading}}{\text{Theoretical loading}}\right)\times 100$$

To analyze the ROS‐responsive degradation properties of TKNP, TEM was used to monitor the change in the morphology and size following incubation under oxidative stress conditions. Briefly, TKNP was incubated in PBS (pH 7.4) supplemented with H_2_O_2_ (0.1 mM) for 0, 6, and 12 h. Next, the size and morphological features were observed under TEM.

### 4.3 Tailoring of anti‐PD‐L1 antibody on TKNP using DPEMA

DPEMA used as a surfactant during TKNP synthesis provides an active site for the conjugation of anti‐PD‐L1 antibody.^15^ To conjugate anti‐PD‐L1 antibody (Bio X Cell, Lebanon, NH), TKNP (1 mg) was incubated with different concentrations of anti‐PD‐L1 antibody (30, 60, 90, 120, and 150 μg/ml) suspended in NaHCO_3_ buffer (pH 8.5). Following incubation for 1 h, repeated purification was conducted at 15,000 × *g* for 10 min at 4°C. To determine the conjugated amount of antibody on TKNP surface, Pierce BCA protein assay kit (Thermo Fisher Scientific) was used. CE were calculated using the formulae:

(3) $$\mathrm{CE}\left(\%\right)=\left(\frac{\text{Amount of anti}-\mathrm{PD}-L1\;\text{antibody conjugated to TKNP}}{\text{Initial amount of anti}-\mathrm{PD}-L1\;\text{antibody used}}\right)\times 100$$

Similarly, DLS was used to monitor the mean hydrodynamic diameter, PDI, and zeta potential of the anti‐PD‐L1‐TKNP.

### 4.4 Oxidative stress‐mediated release of DOX and R848

To assess the release of DOX and R848 from anti‐PD‐L1‐TKNP, anti‐PD‐L1‐DOX/TKNP (1 mg) and anti‐PD‐L1‐R848/TKNP (1 mg) were suspended in PBS (1 ml) maintained at pH 7.4 and 5.0. For the ROS‐responsive release phenomenon, anti‐PD‐L1‐DOX/TKNP and anti‐PD‐L1‐R848/TKNP were suspended in PBS (1 ml, pH 5.0) containing H_2_O_2_ (0.1 mM). Next, the samples were placed in a shaking incubator at 37°C. At certain time intervals, samples were taken and centrifuged at 15,000 × *g* for 10 min at 4°C. The supernatant was examined for the release of DOX and R848 using fluorescence spectrometer and UV spectroscopy, respectively.

### 4.5 Cellular internalization of nanoparticles

MDA‐MB‐231 and BT‐20 cells (Korean Cell Line Bank, Seoul, South Korea) were cultured in RPMI 1640 medium (RPMI, GIBCO, Grand Island, NY) supplemented with fetal bovine serum (FBS), 1% (v/v) penicillin–streptomycin, and 2% (v/v) sodium pyruvate (Gibco‐Invitrogen, Grand Island, NY). The 4T1 cells, provided as a gift from the Seoul National University, were cultured in DMEM medium supplemented with FBS, 1% (v/v) penicillin–streptomycin, and 2% (v/v) sodium pyruvate.

Briefly, MDA‐MB‐231 cells (2 × 10^5^ cells/6‐well plate) pretreated with interferon‐gamma (IFN‐γ, BioLegend) were further incubated with TKNP, anti‐PD‐L1‐TKNP, and anti‐PD‐L1 antibody at 4°C for 2 h. Subsequently, cells were incubated with a secondary antibody (Alexa Fluor 488 anti‐rat IgG, Thermo Fisher Scientific) for 1 h. Analysis was performed using FACS (BD Biosciences, San Jose, CA).

MDA‐MB‐231 and BT‐20 cells pretreated with IFN‐γ were seeded at a density of 2 × 10^5^ cells/6 well. Cells were treated with free coumarin‐6, coumarin‐6‐loaded DOX/TKNP, and anti‐PD‐L1‐DOX/TKNP for 6 h. Subsequently, cells were washed twice with PBS and eventually analyzed using FACS. For qualitative analysis, 2 × 10^5^ MDA‐MB‐231 and BT‐20 cells were seeded onto a coverslip attached to a 6‐well plate. Next, the cells were treated with free coumarin 6, coumarin 6‐loaded DOX/TKNP, and anti‐PD‐L1‐DOX/TKNP for 6 h. Cells were then fixed using 4% paraformaldehyde and counterstained with DAPI (Sigma‐Aldrich) and lysotracker (Thermo Fisher Scientific). Finally, cells were imaged using CLSM (Leica Microsystem, Wetzlar, Germany).

### 4.6 Cellular internalization of DOX and nanoparticles in 3D spheroid model

AggreWell method (STEMCELL Technologies, Vancouver, BC, Canada) was employed to prepare a 3D spheroid model. Briefly, 2.4 × 10^5^ cells/well suspended in a medium containing 2.5% Matrigel were added to each well. Cells were left overnight in AggreWell for 1 day. Next, the spheroids were transferred to 6‐well plate and further incubated with free coumarin 6, coumarin 6‐loaded DOX‐TKNP and anti‐PD‐L1‐DOX‐TKNP for 6 h. Spheroids were then washed twice with PBS and imaged under CLSM.

### 4.7 Measurement of intracellular ROS production

MDA‐MB‐231 and BT‐20 cells (2 × 10^5^) plated in a 6‐well plate were exposed to IFN‐γ and CoCl_2_ (100 μM) for 12 h. Cells were then treated with TKNP, DOX, DOX/TKNP, and anti‐PD‐L1‐DOX/TKNP for 24 h. Subsequently, cells were stained with DCFH‐DA dye (Sigma‐Aldrich Corp.) for measuring intracellular ROS production. Cells were then washed twice with PBS and eventually analyzed using FACS.

### 4.8 In vitro ICD measurement

CRT exposure on the cell surface, intracellular HMGB1 distribution, and extracellular release of ATP were measured to evaluate the ICD phenomenon. Briefly, MDA‐MB‐231 and BT‐20 cells (2 × 10^5^) prior treated with IFN‐γ were subjected to CoCl_2_ (100 μM) for 12 h. Next, the cells were treated with TKNP, DOX, DOX/TKNP, and anti‐PD‐L1‐DOX/TKNP for 24 h. Subsequently, cells were washed twice with PBS. For CRT expression, cells were stained with Alexa fluor 488 conjugated CRT (Cell Signaling Technology, Danvers, MA) dye for 2 h and eventually analyzed using FACS. Qualitative analysis of CRT and HMGB1 was performed using CLSM. For this, 2 × 10^5^ MDA‐MB‐231, BT‐20, and 4T1 cells were plated in a 6‐well plate containing a coverslip. Next, cells were treated with TKNP, DOX, DOX/TKNP, and anti‐PD‐L1‐DOX/TKNP for 24 h. Cells were then washed and incubated with Alexa fluor 488 conjugated CRT dye (Cell Signaling Technology) for 2 h. For HMGB1 staining, cells were incubated with HMGB1 primary antibody (Cell Signaling Technology) followed by incubation with Alexa fluor 488 conjugated secondary antibodies for 1 h. Finally, cells were fixed with 4% formaldehyde and imaged using CLSM.

Extracellular secretion of ATP was tested using ATP assay kit (Abcam, Cambridge, UK). All cells were pretreated with IFN‐γ and CoCl_2_ for 12 h, and were plated in a 96‐well plate at a density of 10^4^ cells/well. Subsequently, cells were treated with TKNP, DOX, DOX/TKNP, and anti‐PD‐L1‐DOX/TKNP for 24 h. According to the manufacturer's instructions, ATP level was measured using a luminescent ATP detection assay kit, and luminescence was measured using a microplate reader.

### 4.9 In vitro assessment of cytotoxicity

To examine the cytotoxic effect, MDA‐MB‐231, BT‐20, and 4T1 cells were seeded in a 96‐well plate (10^4^ cells/well) prior treated with IFN‐γ and CoCl_2_ for 12 h. Cells were further incubated with TKNP, DOX, DOX‐TKNP, and anti‐PD‐L1‐DOX‐TKNP at an equivalent concentration of DOX (1 μg/ml) for 24 h. Subsequently, cells were washed with PBS and cell viability was measured using CCK‐8 assay.

### 4.10 Transwell chemotaxis assay

A solution of free MIP‐3α and anti‐PD‐L1‐MIP‐3α‐TKNP containing equivalent concentration of MIP‐3α (1–1000 ng/ml) was distributed in the lower chamber of a transwell permeable 24‐well plate (12 × 6.5 mm inserts; 8.0 μm PET membrane, Costar Data Packaging Corporation., Cambridge, MA). Next, RAW 264.7 macrophages (1 × 10^4^ cells/well) seeded in the upper chamber of the transwell plate were allowed to migrate for 24 h, following which, cells migrated in the lower chamber were fixed with 4% paraformaldehyde, and optical images were obtained using a fluorescence microscope. For quantitative analysis, cells were stained with methylene blue and manually counted using a microscope.

### 4.11 In vitro assessment of DCs activation and maturation assay

Bone marrow‐derived DCs were isolated from the bone marrow of 8‐week‐old balb/c mice. Immature DCs (5 × 10^5^ cells/well) plated in a 12‐well plate were treated with TKNP, free R848, and anti‐PD‐L1‐R848/TKNP with the equivalent concentration of R848 (30 μmol/L) for 24 h. Cells were washed with PBS, harvested, and stained with PE‐CD11c, FITC‐CD86, and PerCP‐MHC II (BioLegend) and sorted using FACS.

### 4.12 Accumulation and retention of nanoparticles in tumor tissue

Balb/c mice (female; aged 6–8 weeks) were purchased from Hyochang Science Co. Ltd., (Daegu, South Korea). The animal handling and experiments were performed according to the guidelines of the Institutional Animal Ethical Committee, Keimyung University, South Korea. 4T1 tumor‐bearing balb/c mice were injected intratumorally with free Cy5.5, Cy5.5‐loaded TKNP, and anti‐PD‐L1‐TKNP. In vivo imaging was performed using the IVIS (Vieworks, Gyeonggi do, South Korea) at 0, 0.5, 6, 12, and 24 h post‐injection. After 24 h, mice were sacrificed and ex vivo imaging of heart, lungs, liver, spleen, kidney, and tumor were performed.

### 4.13 In vivo antitumor study

The Supporting Information provides the method for preliminary antitumor study for R848 dose optimization. Female Balb/c mice were randomly divided into groups, and were then administered 4T1 cells (1 × 10^5^) suspended in free RPMI medium by subcutaneous injection in the right thigh. PBS, DOX, R848, DOX‐R848‐MIP‐3α, and anti‐PD‐L1‐DOX‐R848‐MIP‐3α/TKNP with equivalent concentration of R848 (1 mg/kg) were injected intratumorally when the tumor size reached 200 mm^3^. The formulation was injected on days 0, 5, 10, and 15. Tumor volumes were determined using a digital caliper by measuring the tumor's major (A) and minor (B) axes. Tumor volumes (*V*) were calculated as follows: *V* = A × B^2^/2. Mice body weights were also measured on certain days.

### 4.14 In vivo maturation of DCs

To examine the DC maturation, mice were treated with different formulations on days 0, 5, and 10. On day 15, mice were sacrificed. Lymph nodes were isolated, digested with collagenase D (1 mg/ml; STEMCELL Technologies) and RNAse (10 μg/ml; Roche Diagnostics, Basel, Switzerland) at 37 °C for 30 min. Next, the cell suspensions were passed through the filter (40‐μm filter, BD Bioscience, Franklin Lakes, NJ). Finally, cells were stained with PE‐CD11c, FITC‐CD86, and PerCP‐MHC II and sorted using FACS.

### 4.15 Intratumoral retention of DOX and expression of ICD marker in tumor tissue

4T1‐tumor‐bearing balb/c mice were treated with different formulations on days 0, 5, and 10. On days 15 and 20, mice were sacrificed, and tumors were collected. Tumors were sectioned (30 μm) and fixed with 4% (v/v) formaldehyde for 15 min followed by incubation with 0.5% (w/v) Triton X‐100 (Acros Organics, Morris Plains, NJ). Subsequently, tumor sections were blocked with 2% (v/v) bovine serum albumin. Tumor slices were then incubated with Alexafluor 488 conjugated CRT dye for 2 h. Tumor sections on day 20 were further stained with HMGB1 antibody overnight at 4°C followed by incubation with Alexafluor 488 dye. Next, tissues were further stained with DAPI for nucleus staining, mounted in a vectashield mounting media (Vector Laboratories, Burlingame, CA), and imaged using confocal microscopy.

### 4.16 Infiltration of CD4 ^+^ and CD8 ^+^ T cells in the tumor microenvironment

To assess the intratumoral infiltration of CD4^+^ and CD8^+^ T cells, tumor cells were digested with RNAse (Roche Diagnostics) and collagenase D (STEMCELL Technologies) to obtain single‐cell suspensions. Next, cells were stained with anti‐CD3‐APC, anti‐CD4‐FITC, and anti‐CD8‐PerCP‐Cy7.7 (BioLegend, San Diego, CA) for 15 min in ice. Cells were then washed twice with PBS and sorted using FACS caliber flow cytometer.

Furthermore, CD4^+^ and CD8^+^ T cell expression levels in the tumor tissue were further examined using immunofluorescence staining. Tumors sections (20 μm) were prepared using a microtome (Leica Biosystem, Seoul, South Korea), blocked with bovine serum albumin, and fixed with 4% paraformaldehyde. Subsequently, tissue sections were further stained with anti‐CD4‐FITC and anti‐CD8‐PE (BioLegend, San Diego, CA) and incubated for 30 min at 4°C. Cells were then washed with PBS, mounted with vectashield mounting, counterstained with DAPI, and imaged using CLSM.

### 4.17 Detection of cytokine level in the tumor microenvironment and serum

Activated CD8^+^ T cells secrete cytokines that exert antitumor effect. Therefore, we further assessed cytokine levels in the tumor microenvironment and serum. For cytokine measurement, single‐cell suspensions of the tumor were plated in a 96‐well plate. Cells were stimulated with phorbol‐12‐myristate‐13‐acetate, ionomycin, and Golgi transport inhibitor for 4 h in the incubator at 37°C. Subsequently, cells were stained with anti‐CD3‐APC, anti‐CD4‐FITC, and anti‐CD8‐PerCP‐Cy7.7 conjugated CD8 antibody for 15 min. Following incubation, cells were washed, fixed with 4% formaldehyde, and further stained with granzyme PE and perforin PerCP‐Cy5.5 (BioLegend, San Diego, CA) for 15 min. Cells were then washed twice with PBS and analyzed with FACS.

Additionally, serum cytokine level was measured on day 15 and 20. Cytokines such as TNF‐α and IL‐6 were evaluated using a TNF‐α ELISA kit (BioLegend, San Diego, CA) and an IL‐6 Elisa kit (BD Biosciences) as per the manufacturer's instructions.

### 4.18 Statistical analysis

Data were expressed as mean ± SD, and statistical significance was measured using Student's two‐tailed *t*‐test. Statistical significance was set at *p* < 0.05.

## 5 CONCLUSIONS

We reported a novel synthesis of PD‐L1‐targeted ROS‐responsive TKNPs for combination chemoimmunotherapy. The anti‐PD‐L1‐DOX‐R848‐MIP‐3α/TKNP provided better tumor specificity due to the construction of tumor (PD‐L1) and tumor‐microenvironment (ROS)‐responsive nanosystem. The nanosystem effectively causes enhanced intratumoral infiltration of immune cells. The combination of chemotherapy, immunoadjuvant, and anti‐PD‐L1‐DOX‐R848‐MIP‐3α/TKNP enhanced ICD and caused maximal maturation of DCs. Furthermore, anti‐PD‐L1‐DOX‐R848‐MIP‐3α/TKNP‐treated mice showed increased intratumoral infiltration of CD4^+^ and CD8^+^ T cells and enhanced level of circulating and tumor‐infiltrated cytokines for better antitumor effect. Thus, combination therapy of chemokines, immunoadjuvants, and cytotoxic agents in the dual‐targeted and tumor microenvironment‐responsive nanosystem could be an effective strategy for better anticancer therapy.

## AUTHOR CONTRIBUTIONS

**Asmita Banstola**: Conceptualization; data curation; investigation; formal analysis; project administration; validation; visualization; writing – original draft preparation; writing – review & editing. **Mahesh Pandit**: Investigation; formal analysis; visualization. **Ramesh Duwa**: Investigation; formal analysis; visualization. **Jae‐Hoon Chang**: Conceptualization; writing – review & editing. **Jee‐Heon Jeong**: Conceptualization; project administration; supervision; writing – original draft preparation; writing – review & editing. **Simmyung Yook:** Conceptualization; data curation; project administration; validation; supervision; writing – original draft preparation; writing – review & editing.

## CONFLICT OF INTEREST

The authors have no conflicts of interest to declare.

### PEER REVIEW

The peer review history for this article is available at https://publons.com/publon/10.1002/btm2.10379.

## Supporting information


**Appendix S1** Supporting information

## Data Availability

All data needed to evaluate the conclusions in the paper are present in the paper and/or the Supplementary Materials. Additional data related to this paper may be requested from the authors.
