# Supplementary material for: Reactive oxygen species‐responsive dual‐targeted nanosystem promoted immunogenic cell death against breast cancer
Source: Bioeng Transl Med. 2022 Aug 3;8(5):e10379. doi: 10.1002/btm2.10379 (PMC10487313; doi:10.1002/btm2.10379)
Supplement: Supplementary file 1 — Appendix S1 Supporting information [file BTM2-8-e10379-s001.docx]

**Supporting Information.** Detailed experimental section and result section; Figure S1, Loading and encapsulation efficiency of DOX, R848 and MIP-3α in TKNP; Figure S2, Zeta potential measurement; Figure S3, Particle size and zeta potential measurement for the optimization of different concentration of anti-PD-L1 antibody conjugated on the surface of TKNP; Figure S4, Stability of anti-PD-L1-DOX-R848-MIP-3α/TKNP in PBS after 7 days; Figure S5, Flow cytometry analysis for the evaluation of retention of immunoreactivity of antibody after conjugation on TKNP; Figure S6, CLSM images showing cellular internalization of nanoparticles and DOX in MDA-MB-231 cell lines 3D after 4 h incubation with DOX-TKNP, anti-PD-L1-DOX-TKNP and anti-PD-L1 pretreatment + anti-PD-L1-DOX-TKNP; Figure S7, Assessment of cell cytotoxicity effect following treatment with different concentration of CoCl2 (µM) in MDA-MB-231 and BT-20 cell line for 12 h; Figure S8, Western blot assay for the evaluation of hypoxia (HIF-1α) following treatment with different concentration of CoCl2 (µM) in MDA-MB-231 and BT-20 cell line; Figure S9, FACS analysis for the determination of total ROS production after the treatment of different concentration of CoCl2 for 12 h in MDA-MB-231 and BT-20 cell line; Figure S10, Measurement of serum IL-6 level following intra-tumoral injection of different concentration of R848 encapsulated in anti-PD-L1-DOX-R848-MIP-3α/TKNP; Figure S11, Gating strategy for CD4 T cells, CD8 T cells, granzyme and perforin in the tumor tissue (PDF).

*Supporting Information*

**Reactive Oxygen Species-Responsive Dual Targeted Nanosystem Promoted Immunogenic Cell Death Against Breast Cancer**

Asmita Banstola^1,2^, Mahesh Pandit^3^, Ramesh Duwa^1^, Jae-Hoon Chang^3^, Jee-Heon Jeong^4,*^, Simmyung Yook^1, *^

^1^College of Pharmacy, Keimyung University, Daegu 42601, South Korea

*^2^Wellman Center for Photomedicine, Massachusetts General Hospital, Department of Dermatology, Harvard Medical School, Boston, MA 02114, USA*
^3^College of Pharmacy, Yeungnam University, Gyeongsan, Gyeongbuk 38541, South Korea

^4^Department of Precision Medicine, School of Medicine, Sungkyunkwan University, Suwon 16419, South Korea

*Corresponding Authors

**Simmyung Yook, Ph.D.**

College of Pharmacy, Keimyung University, *Daegu 42601, South Korea*

E-mail: [ysimmyung@kmu.ac.kr](mailto:ysimmyung@kmu.ac.kr); Tel: +82–53–580–6656

**Jee-Heon Jeong, Ph.D.**

Department of Precision Medicine, School of Medicine, Sungkyunkwan University, Suwon 16419, South Korea

E-mail: [jeeheon@skku.edu](mailto:jeeheon@skku.edu); Tel: + 82-31-299-6165

**ADDITIONAL METHODS**

**Construction and characterization of DOX, MIP-3α and R848 laden thioketal nanoparticles**

Briefly, thioketal polymer (1 mg) was dissolved in dichloromethane (5 mL) containing DOX (Zhejing Hisum Co, Zhejiang, China), R848 (MedChemExpress, MCE, NJ, USA) and MIP-3α (Biolegend, San Diego, CA, USA) in the different mass ratio of (10:1:0.5:0.04, 10:2:0.5:0.04, 10:1:1:0.04 and 10:1:0.5:0.02) respectively. To the mixture, DPEMA {5 mL, 1% (w/v)} was added. Then the solution was probe sonicated (Sonics and Materials, INC., Newtown, USA) for 60 s at 60% amplitude with a probe sonicator. Furthermore, DPEMA {5 mL, 0.1% (w/v)} was added following sonication and stirring was continued overnight. Repeated purification was carried out following centrifugation at 15,000 × g for 10 min to obtain DOX-R848-MIP-3α co-loaded TKNP.

**Stability study of anti-PD-L1-DOX-R848-MIP-3α/TKNP**

We further incubate anti-PD-L1-DOX-R848-MIP-3α/TKNP in PBS for 7 days and measure the particle size and PDI to access the stability of anti-PD-L1-DOX-R848-MIP-3α/TKNP.

**Cell surface binding study**

We further evaluated immunoreactivity of anti-PD-L1 antibody after conjugation with TKNP. Briefly, MDA-MB-231 cells (2 × 10^5^ cells) pretreated with interferon-gamma (IFN-γ, BioLegend, San Diego, CA, USA) were plated in a 6-well plate. Cells were then incubated with different amount of anti-PD-L1 antibody (30 µg, 60 µg, 90 µg, 120 µg and 150 µg) conjugated TKNP for 2 h at 4 ºC. Afterwards, cells were further incubated with secondary antibody (Alexa Fluor 488 anti-rat IgG, Thermo Fisher Scientific, Rockford, IL, USA) for 1 h and detection was done using fluorescence activated cell sorting (FACS, BD Biosciences, CA, USA). Cells treated with TKNP serves as a negative control and cells treated with native anti-PD-L1 antibody serves as positive control.

**Competitive receptor binding assay**

We then performed competitive receptor binding assay to understand the mechanism of cellular internalization of our nanoformulations. MDA-MB-231 3D spheroid cells were treated with coumarin-6 loaded DOX-TKNP, coumarin-6 loaded anti-PD-L1-DOX-TKNP as well as anti-PD-L1 antibody pretreated coumarin-6 loaded anti-PD-L1-DOX-TKNP for 4 h. After 4 h, cells were washed twice with PBS, fixed with formaldehyde (4%) and imaged using using CLSM.

**Measurement of cobalt chloride (CoCl_2_) induced cellular cytotoxicity**

MDA-MB-231 and BT-20 cells at a density of 1 × 10^4^ cells were seeded in a 96 well plate. Afterwards, cells were incubated with different concentration of CoCl_2_ ranging from 25-800 µM for 12 h. Then, cells were washed with PBS and further incubated with 10 µL of CCK-8 solution and viabilty was measure using microplate reader (Tecan, Msing 16 Inf, Switzerland) at 450 nm.

**Measurement of CoCl_2_ induced hypoxia**

MDA-MB-231 cells and BT-20 cells (2 × 10^5^ cells/well) were incubated with different concentration of CoCl_2_ ranging from 25-800 µM for 12 h. After 12 h, cells were washed with PBS, trypsinized and lysed with lysis buffer. The protein concentration were examined using pierce BCA protein assay kit and the extracted proteins were separated using Bis-tris polyacrylamide gel operated at 100 V for 90 min. Afterwards, proteins were transferred to the membranes and membrane was further incubated with HIF-1α antibody overnight at 4ºC. Then the membranes were further incubated with secondary antibody and imaged using Image Quant LAS 4000 (Taunton, MA, USA) by soaking in a chemiluminescent substrate.

**Determination of hypoxia induced ROS level**

MDA-MB-231 cells and BT-20 cells (2 × 10^5^ cells/well) seeded in a 12 well plate were treated with different concentration of CoCl_2_ for 12 h. After 12 h, cells were incubated with DCFHDA dye and analysis was done using FACS.

**Preliminary antitumor study for dose optimization of R848**

Female balb/c mice, aged 6-8 weeks, were divided into groups at random. We then inject 1 × 10^5^ 4T1 cells suspended in free RPMI medium by subcutaneous injection to the right thigh of balb/c mice. After tumor reached volume of 100 mm^3^, PBS and anti-PD-L1-DOX-R848-MIP-3α/TKNP containing different concentration of R848 (0.1, 1 and 10 mg/kg) was given intratumorally to the mice at 0 and 5 days. Tumor volume was measured with the aid of digital caliper to measure major (A) and minor axes (B) of tumor. Tumor volume was calculated as, V= A × B^2^/2.

**Measurement of serum cytokine level**

*We then measured* cytokine level (IL-6) at day 10 in the serum of the treated mice. The level of IL-6 were evaluated using ELISA kit as per the manufacturer’s instructions.

**ADDITIONAL RESULTS**

**Construction and characterization of DOX, MIP-3α and R848 laden thioketal nanoparticles**

An emulsion-solvent evaporation technique was used for the preparation of DOX, R848 and MIP-3α laden TKNP. The loading capacity and encapsulation efficiency of DOX, R848 and MIP-3α in the mass ratio of TKNP:DOX:R848:MIP-3α (10:1:0.5:0.04) was found to be (3.6 ± 0.1%; 35.9 ± 1.2%, 3.8 ± 0.3%; 76.8 ± 6.9%, and 0. 3 ± 0.03%; 91.9 ± 9.6%) respectively (Figure S1). Similarly, loading capacity of DOX, R848 and MIP-3α in the mass ratio of TKNP:DOX:R848:MIP-3α (10:2:0.5:0.04) was found to be (4.2 ± 0.2%; 21.2 ± 0.9%, 2.8 ± 0.2%; 56.9 ± 5.6%, and 0.2 ± 0.03%; 68.2 ± 9.1%) respectively. Furthermore, the loading capacity of DOX, R848 and MIP-3α in the mass ratio of TKNP:DOX:R848:MIP-3α (10:1:1:0.04) was found to be (3.4 ± 0.2%; 34.2 ± 2.0%, 4.1 ± 0.4%; 40.8 ± 4.2%, and 0.3 ± 0.02%; 77.7 ± 7.0% respectively. Additionally, the loading capacity and encapsulation efficiency of DOX, R848 and MIP-3α in the mass ratio of TKNP:DOX:R848:MIP-3α (10:1:0.5:0.02) was found to be (3.2 ± 0.3%; 32.3 ± 3.2%, 3.4 ± 0.2%; 69.6 ± 5.2%, and 0.1 ± 0.01%; 93.6 ± 7.2%) respectively. Overall, we selected TKNP:DOX:R848:MIP-3α with the mass ratio of (10:1:0.5:0.04) for effective loading of DOX, R848 and MIP-3α.

We also measured the zeta potential of different mass ratio of TKNP:DOX:R848:MIP-3α. Our result demonstrated that TKNP:DOX:R848:MIP-3α in the mass ratio of (10:1:0.5:0.04), (10:2:0.5:0.04), (10:1:1:0.04) and (10:1:0.5:0.02) exhibit surface charge of -33.3 ± 1.2 mV, -31.6 ± 0.9 mV, 32.1 ± 1.9 mV and 35.2 ± 1.9 mV respectively (Figure S2).

**Characterization of anti-PD-L1 antibody conjugated thioketal nanoparticles**

We then measured the particle size and zeta potential following different amount of anti-PD-L1 antibody conjugated on TKNP (Figure S3). Our result demonstrated that anti-PD-L1-TKNP (30 µg/mL) showed particle size of 98.9 ± 2.2 nm; PDI-0.31 and zeta potential of -31.2 ± 2.3 mV. Similarly, following conjugation with 60 µg/mL of anti-PD-L1-TKNP, particle size was 99.4 ± 3.0 nm; PDI-0.37 and zeta potential of -27.5 ± 1.2 mV. After conjugation with 90 µg/mL of anti-PD-L1 antibody on TKNP, we observed particle size of 100.2 ± 1.4 nm; PDI-0.30 and zeta potential of -13.0 ± 2.9 mV. Furthermore, conjugation with 120 µg/mL of anti-PD-L1 antibody on TKNP caused increment in the particle size to 103.3 ± 3.0 nm; PDI-0.28 and zeta potential of -10.5 ± 0.3 mV. However, conjugation of 150 µg/mL of anti-PD-L1 antibody further caused increment in particle size to 118.2 ± 3.1 nm; PDI-0.33 and zeta potential of -7.0 ± 0.9 nm. Thus, from physicochemical characterization study, we observed that following conjugation with anti-PD-L1 antibody, there was increase in the particle size and positive shift of zeta potential.

**Cell surface binding study**

After confirming the conjugation of antibody, we evaluated the MDA-MB-231 cell surface binding ability of different amount of anti-PD-L1 antibody conjugated on TKNP using flow cytometry (Figure S5). Our result demonstrated that compared the binding affinity of 120 ug/mL of antibody used for conjugation on TKNP was similar to the positive control (free anti-PD-L1 antibody). However, when 150 ug/mL of antibody was used for conjugation on TKNP, there was decreased in binding affinity. Therefore, we selected 120 ug/mL of antibody used for conjugation on TKNP.

**Determination of cytotoxicity of cobalt chloride (CoCl_2_)**

To generate hypoxia in cancer cells, we used CoCl_2_ (Figure S7). Since CoCl_2_ is well reported for induction of hypoxia, we measured cell viability with different concentration of CoCl_2._ Our result demonstrated that CoCl_2_ with concentration less than 200 µM were non-toxic to both MDA-MB-231 and BT-20 cell line. However, when cells were incubated with increased concentration of CoCl_2_, there was reduction in the cell viability in both MDA-MB-231 and BT-20 cell line suggesting concentration dependent cell cytotoxic effect of CoCl_2_.

**Tumor hypoxia development and ROS measurement**

After confirming non cytotoxic effect of CoCl_2_, we then measure expression of HIF-1α in MDA-MB-231 and BT-20 cell line following treatment with CoCl_2_ upto concentration of 200 µM (Figure S8). Our result demonstrates that there was concentration dependent increment in expression of HIF-1α with maximum HIF-1α expression observed at concentration of 200 µM.

It is well reported that hypoxic condition accelerates ROS production in cancer cells. Since, our ROS-responsive TKNP works better in high ROS condition, we measured ROS level following treatment with different concentration of CoCl_2_ (Figure S9). Our result demonstrates that there was maximum ROS production in 100 µM of CoCl_2_. The ROS level of 100 and 200 µM of CoCl_2_ was similar in both MDA-MB-231 and BT-20 cell line. Therefore, we used 100 µM of CoCl_2_ for our further experiments as it generates hypoxic condition and high ROS level in breast cancer cell lines.

**Measurement of serum cytokine level**

Following preliminary antitumor study, we evaluated serum IL-6 level in the mice treated with different concentration of R848 (Figure S10). Our result demonstrates that there was concentration dependent increment in serum IL-6 level following treatment with different concentration of R848 encapsulated in anti-PD-L1-DOX-MIP-3α/TKNP. However there was not significant difference in serum IL-6 level in the mice treated with anti-PD-L1-DOX-R848-MIP-3α/TKNP ( 1 mg/kg R848; 140.4 ± 34 pg/mL) compared to the mice treated with anti-PD-L1-DOX-R848-MIP-3α/TKNP (10 mg/kg R848; 151.8 ± 26 pg/mL). Thus, we chose anti-PD-L1-DOX-R848-MIP-3α/TKNP (1 mg/kg R848) for our further *in vivo* experiments.

**
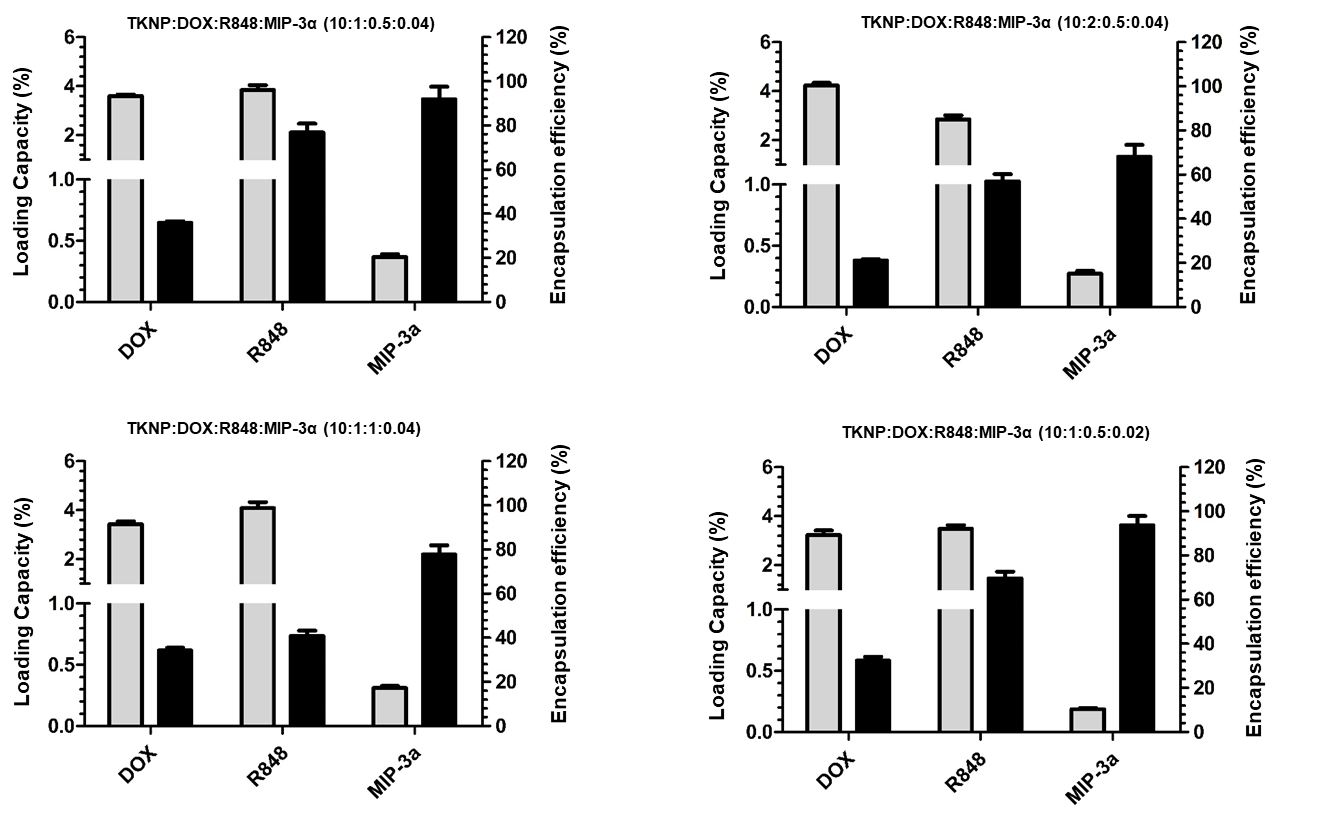
**

**Figure S1.** Optimization of the loading capacity and encapsulation efficiency of the different ratio of DOX, R848 and MIP-3α in TKNP.


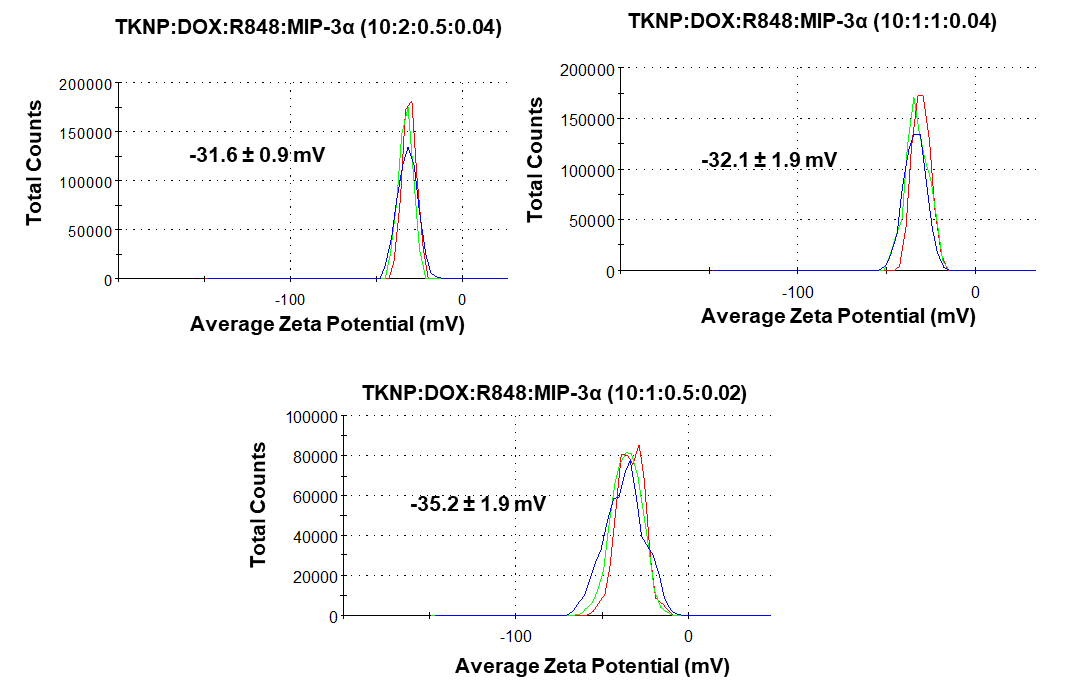


**Figure S2.** Zeta potential measurement for the optimization of different ratio of DOX, R848 and MIP-3α in TKNP.


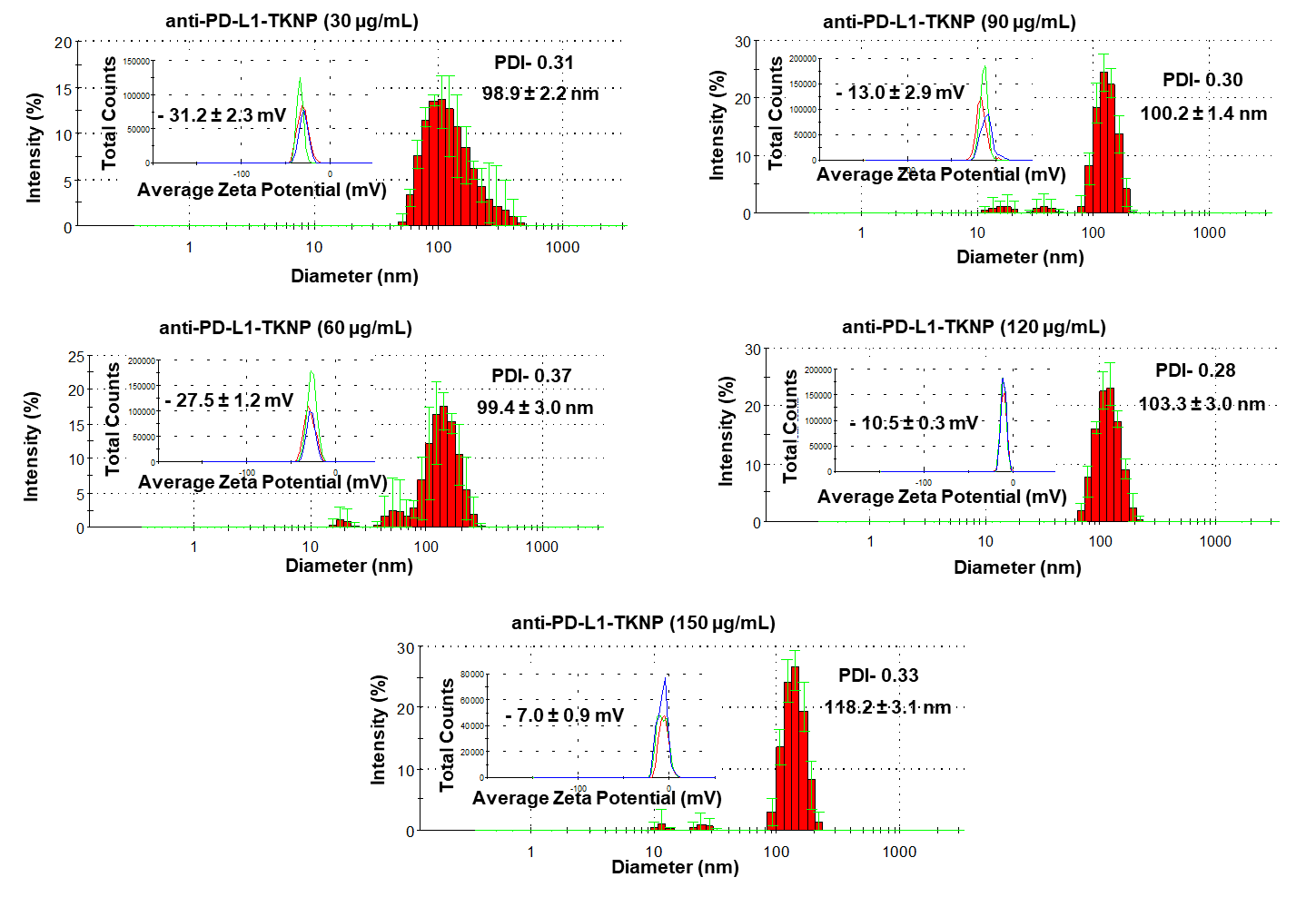


**Figure S3.** Particle size and zeta potential measurement for the optimization of different concentration of anti-PD-L1 antibody conjugated on the surface of TKNP.


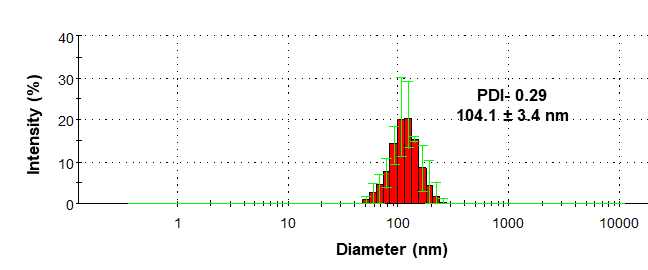


**Figure S4.** Stability of anti-PD-L1-DOX-R848-MIP-3α/TKNP in PBS after 7 days.


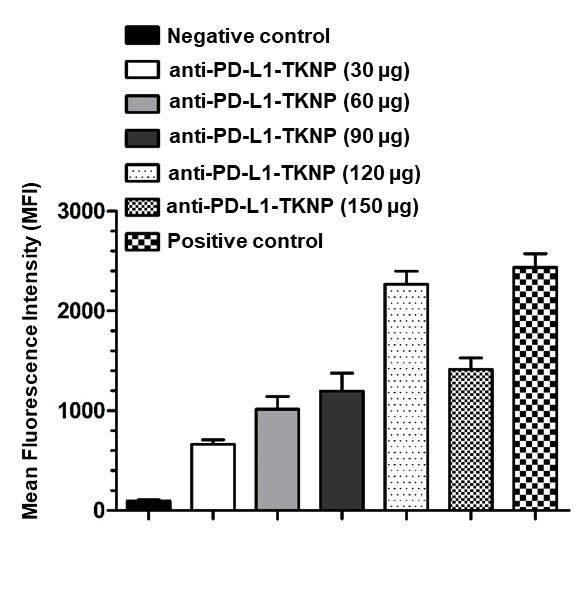


**Figure S5.** Flow cytometry analysis for the evaluation of retention of immunoreactivity of antibody after conjugation on TKNP.


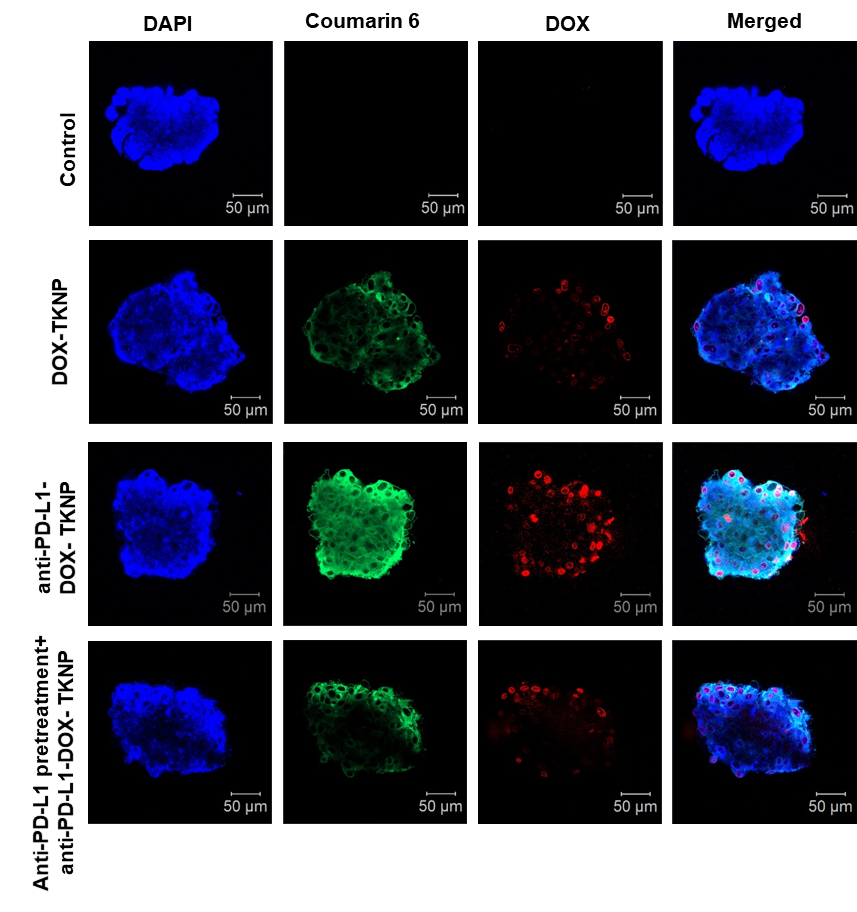


**Figure S6.** CLSM images showing cellular internalization of nanoparticles and DOX in MDA-MB-231 cell lines 3D after 4 h incubation with DOX-TKNP, anti-PD-L1-DOX-TKNP and anti-PD-L1 pretreatment + anti-PD-L1-DOX-TKNP.

**
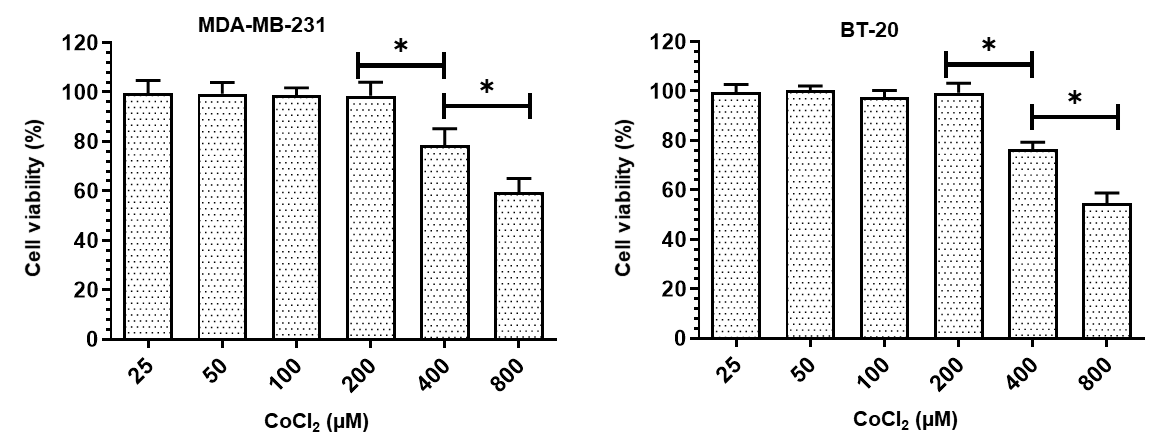
**

**Figure S7.** Assessment of cell cytotoxicity effect following treatment with different concentration of CoCl_2_ (µM) in MDA-MB-231 and BT-20 cell line for 12 h.

**
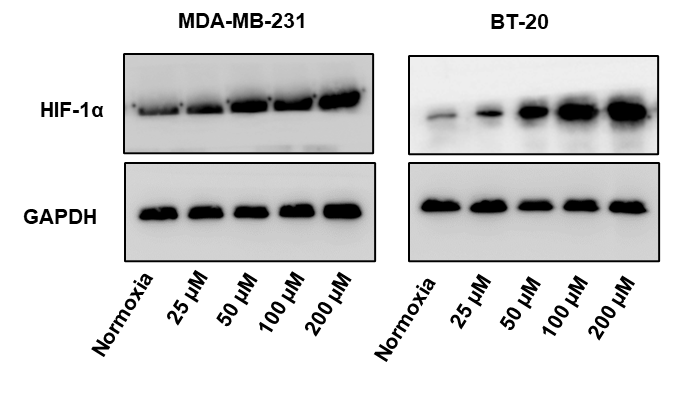
**

**Figure S8.** Western blot assay for the evaluation of hypoxia (HIF-1α) following treatment with different concentration of CoCl_2_ (µM) in MDA-MB-231 and BT-20 cell line.

**
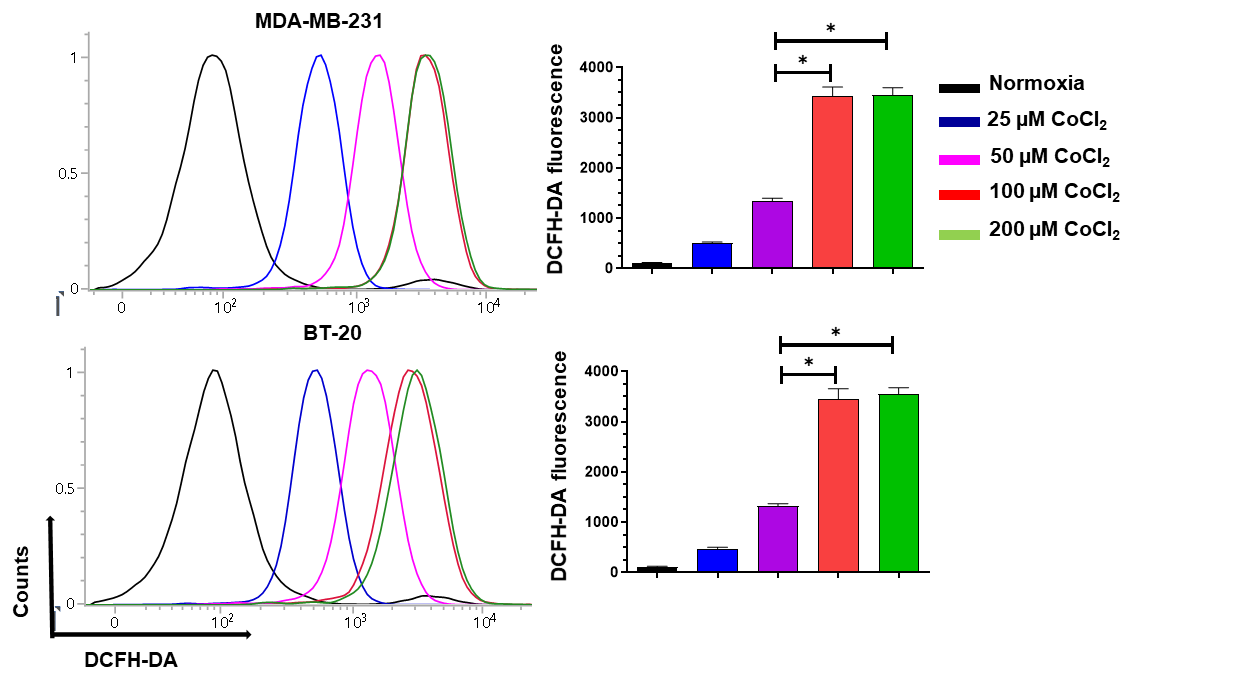
**

**Figure S9.** FACS analysis for the determination of total ROS production after the treatment of different concentration of CoCl_2_ for 12 h in MDA-MB-231 and BT-20 cell line.

**Figure S10.** Measurement of serum IL-6 level following intra-tumoral injection of different concentration of R848 encapsulated in anti-PD-L1-DOX-R848-MIP-3α/TKNP.


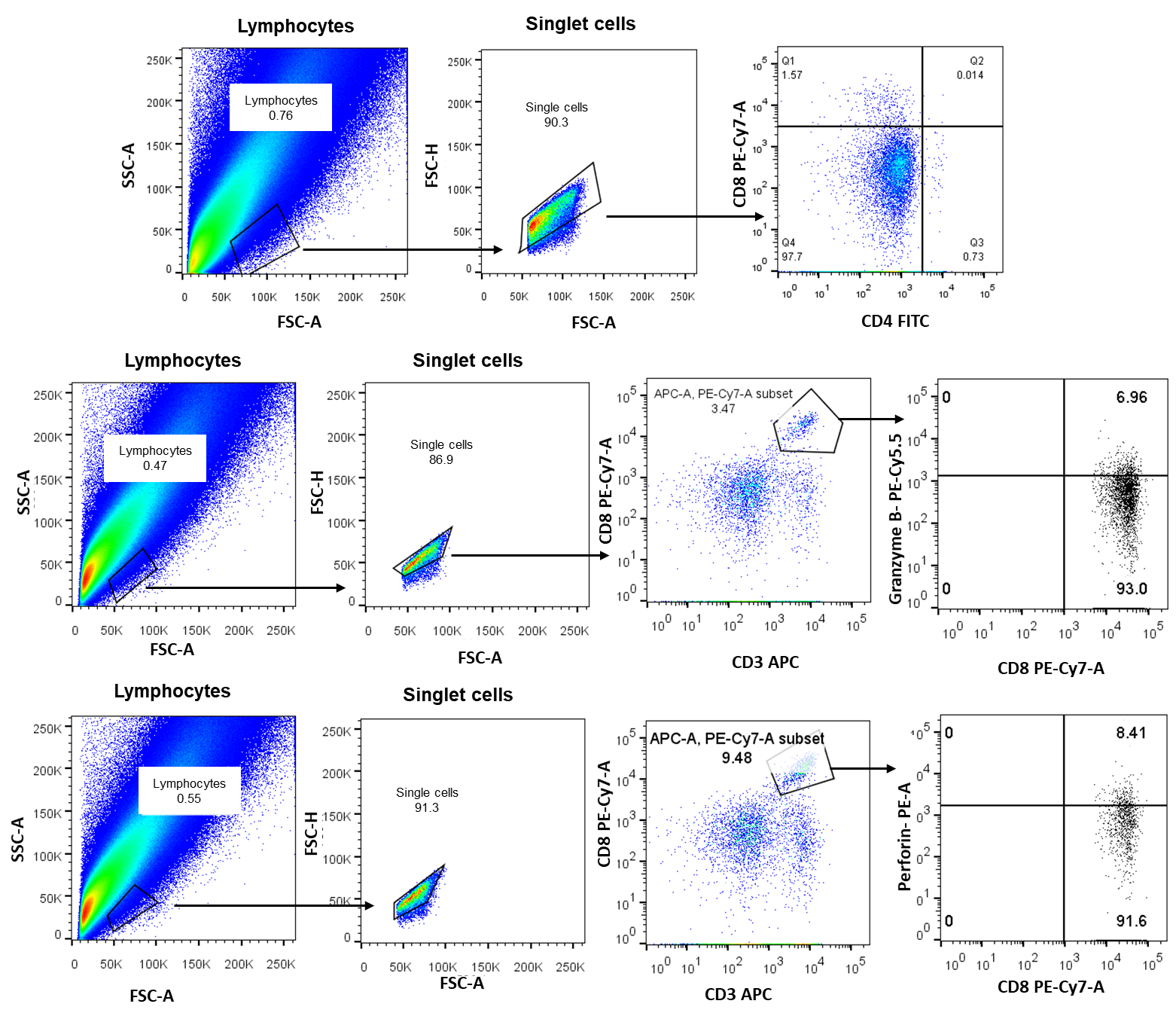


**Figure S11.** Gating strategy for CD4 T cells, CD8 T cells, granzyme and perforin in the tumor tissue.
